# BLOC1S1 Attenuates *B. Melitensis* 16M LPS‐Triggered Autophagy by Spatial Confinement of TDP‐43

**DOI:** 10.1002/advs.202505635

**Published:** 2025-09-11

**Authors:** Shicheng Wan, Miao Han, Mengfei Zhang, Wenbo Chen, Fangde Xie, Xuan Luo, Wenping Wu, Congliang Wang, Donghui Yang, Bin Han, Haijing Zhu, Haisheng Yu, Na Li, Jinlian Hua

**Affiliations:** ^1^ College of Veterinary Medicine Shaanxi Centre of Stem Cells Engineering & Technology Northwest A&F University Yangling Shaanxi 712100 China; ^2^ Guangzhou Eighth People's Hospital Guangzhou Medical University Guangzhou 510440 China; ^3^ College of Veterinary Medicine Xinjiang Agricultural University Urumqi 830000 China; ^4^ Laboratory of Animal Disease Model College of Veterinary Medicine Sichuan Agricultural University Chengdu 611130 China; ^5^ Shaanxi Provincial Engineering and Technology Research Center of Cashmere Goats Life Science Research Center Yulin University Yulin 719000 China

**Keywords:** autophagy, BLOC1S1, *Brucella* spp, LPS, TDP‐43

## Abstract

Biogenesis of lysosome‐related organelles complex 1 subunit 1 (BLOC1S1) is considered to have anti‐*Brucella* potential. However, the effect of BLOC1S1 on *Brucella* autophagy has not yet been studied. This study investigates the interplay between *Brucella* lipopolysaccharide (LPS) and BLOC1S1 in modulating autophagy within goat spermatogonial stem cells (mGSCs‐I‐SB). Using LPS from *B. melitensis* 16M, its capacity is demonstrated to induce AMPK‐dependent autophagy, contrasting with *Escherichia coli* LPS, which shows no significant effect. Mechanistically, *B. melitensis* 16M LPS activates AMPK signaling, elevates LC3B‐II/LC3B‐I ratios, and upregulates lysosomal and pro‐inflammatory genes. BLOC1S1 overexpression attenuates autophagy, reducing autolysosome formation (TEM) and LC3B‐II/I ratio. RNA sequencing and proteomic analyses reveal BLOC1S1‐mediated transcriptional reprogramming of lysosomal pathways and mitochondrial metabolism. Co‐immunoprecipitation and subcellular localization studies reveal that TDP‐43 is a key interacting partner and that BLOC1S1 sequesters TDP‐43 in the cytoplasm, inhibiting its nuclear translocation‐dependent *ATG7* mRNA stability and enhancing autophagy. These findings delineate a dual regulatory mechanism: *B. melitensis* 16M LPS‐driven, AMPK‐dependent autophagy induction, and BLOC1S1‐mediated autophagic suppression through spatial control of TDP‐43. These results advance understanding of host‐pathogen interactions in brucellosis and identify BLOC1S1 as a potential therapeutic target for bacterial persistence and TDP‐43‐related pathologies.

## Graphical abstract

This study explores how *B. melitensis* 16M LPS and BLOC1S1 regulate autophagy in goat spermatogonial stem cells. The 16M LPS is activating AMPK‐dependent autophagy, increasing LC3B‐II/I ratios and lysosomal genes, while *Escherichia coli* LPS shows no effect. BLOC1S1 overexpression is suppressing autophagy by reducing autolysosomes and altering mitochondrial metabolism. Researchers are identifying BLOC1S1's interaction with cytoplasmic TDP‐43, which is blocking nuclear *ATG7* mRNA stabilization.

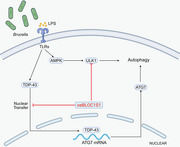

## 1 Introduction

Brucellosis caused by *Brucella* spp. is a severe zoonosis that causes substantial economic losses.^[^ ^1^ ^]^ *Brucella*, a Gram‐negative intracellular pathogen, is classified into smooth and rough types based on the presence or absence of lipopolysaccharide (LPS) O‐chains.^[^ ^2^ ^]^ The main species pathogenic to humans include *B. melitensis* (most virulent), *B. abortus*, *B. suis*, and *B. canis*.

Infection in female animals frequently manifests as miscarriage, stillbirth, or mastitis, whereas infection in male animals often results in orchitis and arthritis, placing a heavy burden on agriculture. Clinical studies have shown that brucellosis presents as acute, subacute, or chronic. Although the mortality rate remains low, the disease often develops into chronic complications and can lead to disability, posing serious threats to human health.^[^ ^3^, ^4^ ^]^

After infection, *Brucella* survives in macrophages by deploying virulence factors and subsequently disseminates hematogenously to multiple tissues, adopting a facultative and stealthy intracellular lifestyle that perpetuates host damage.^[^ ^5^ ^]^ Intracellular replication requires coordinated virulence mechanisms: After phagocytosis, *Brucella* resides in double‐membrane compartments called *Brucella*‐containing vacuoles (BCVs). During initial transport, BCV briefly interact with lysosomes and acquire lysosomal markers (LAMP1, RAB7) to form early BCVs (eBCVs). *β*‐1,2‐glucan inhibits lysosomal fusion. Simultaneously, the Type IV secretion system detects transient acidic signals to secrete effector proteins, allowing eBCVs to migrate to the perinuclear region.^[^ ^6^, ^7^ ^]^

A fraction of BCVs associates with endoplasmic reticulum (ER)‐derived vesicles near the ER‐Golgi interface, acquiring ER markers to establish replicative BCVs (rBCVs) that support bacterial proliferation.^[^ ^8^, ^9^ ^]^ At the late stage of infection, rBCVs transition into autophagic BCVs (aBCVs), Ultimately facilitates the replication of bacteria within the cell.^[^ ^10^, ^11^ ^]^

Manipulation of the host's autophagy‐lysosomal pathway is a central aspect of this intracellular lifestyle. Autophagy is a conserved degradation process characterized by formation of double‐membrane autophagosomes that fuse with lysosomes, thereby clearing intracellular pathogens.

Biogenesis of lysosome‐related organelles complex 1 subunit 1 (BLOC1S1), a multifunctional protein that serves as a subunit of the BORC complex. BORC promote lysosomal trafficking and targeting by recruiting ARL8 GTPase.^[^ ^12^, ^13^, ^14^, ^15^ ^]^ Thus, it controls the efficiency of autophagosome‐lysosome fusion. Deficiency of BLOC1S1 disrupts lysosomal motility and impairs autophagic flux.^[^ ^16^ ^]^ TAR DNA‐Binding Protein 43 (TDP‐43) is an RNA‐binding protein that play an important role in stabilizing *ATG7* mRNA and enhancing autophagosome formation.^[^ ^17^ ^]^ Nuclear TDP‐43 maintains autophagy by promoting *ATG7* expression, whereas its cytoplasmic mislocalization, a hallmark of ALS/FTD, inhibits autophagy and disrupts lysosomal function.^[^ ^18^, ^19^, ^20^ ^]^ In addition to its abnormal accumulation in neurological diseases, TDP‐43 can also accumulate when autophagy is impaired, triggering a vicious cycle of cellular stress and impaired autophagy.^[^ ^21^ ^]^

Lipopolysaccharide (LPS), a major virulence factor of *Brucella*, activates immune‐related gene expression by recognizing TLR4 recognition on host cell membranes. This process occurs through either the MyD88‐dependent pathway or the TRIF‐dependent pathway, the latter of which is initiated after the TLR4/MD‐2/LPS complex is internalized.^[^ ^22^ ^]^ Unlike canonical LPS (e.g., *Escherichia coli* (*E. coli*) LPS), smooth *Brucella* LPS exhibits unique structural modifications: Lipid A: Contains unusually long C16–18 fatty acids (up to C28), whereas the acyl chains in LPS are primarily C12‐14. These longer fatty acids reduce the binding affinity of MD‐2 and endotoxic activity. Core Oligosaccharide: Features two 3‐deoxy‐D‐manno‐oct‐2‐ulosonic acid (Kdo) residues. One Kdo links to the O‐polysaccharide, while the other bears a positively charged side chain. This cationic side chain is hypothesized to exert a “shielding” effect, neutralizing the core's inherent negative charge and preventing MD‐2/TLR4 recognition.^[^ ^2^, ^23^ ^]^ These structural adaptations attenuate host immune detection of smooth *Brucella*, synergizing with other immune evasion strategies to facilitate intracellular persistence.

Considering the role of BLOC1S1 in lysosomal dynamics and TDP‐43 in autophagy initiation, we hypothesized that these molecules constitute critical host defenses against *Brucella* infection. However, their interplay in pathogen‐triggered autophagy remains unexplored. Our study has shown that LPS isolated from *B. melitensis* 16M elicits unique responses in goat spermatogonial stem cells (mGSCs‐I‐SB) compared to *E.coli* LPS. Although neither LPS induced apoptosis, mGSCs‐I‐SB displayed greater sensitivity to *B. melitensis* 16M LPS (16M LPS), which specifically elevated autophagic flux.

This induction of autophagy was mechanistically linked to AMPK activation. Specifically, BLOC1S1 overexpression reduced LPS‐induced autophagy by inhibiting TDP‐43 nuclear translocation. These results explain two important mechanisms: 16M LPS‐driven AMPK‐dependent autophagy potentiation in mGSCs‐I‐SB, and BLOC1S1‐mediated autophagy regulation by regulating TDP‐43 nuclear translocation.

## 2 Results

### 2.1 *Brucella* Infection Downregulates BLOC1S1 Expression in Goat Testes

**Figure 1:**
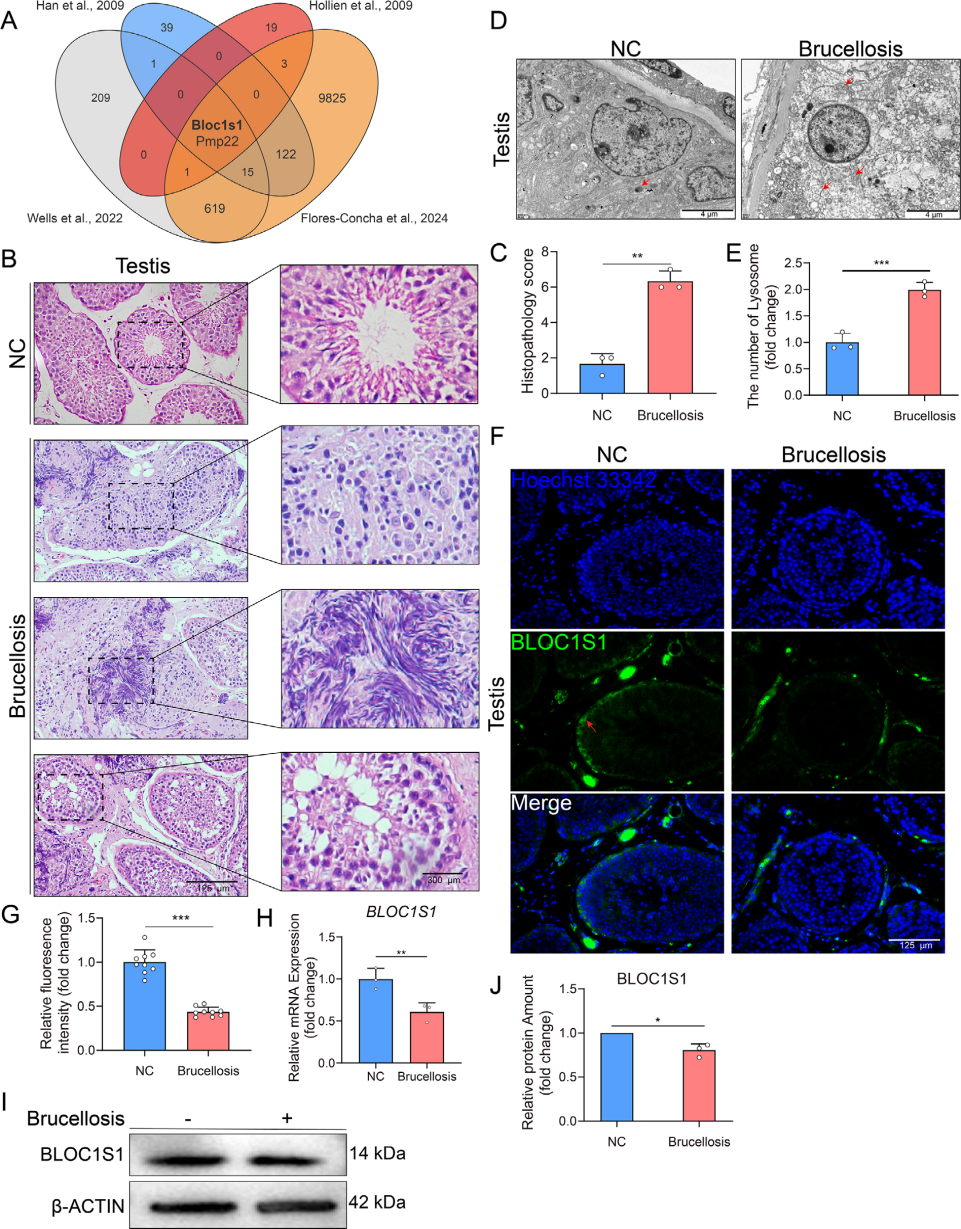
*Brucella* infection downregulates BLOC1S1 expression in goat testes. A) Venn diagram demonstrating RIDD‐targeted genes with reduced expression following *Brucella* infection. B) Hematoxylin and eosin (H&E) staining of testicular sections from *Brucella*‐infected or not‐infected goats. Enlarged regions (top to bottom) highlight characteristic pathological changes: disrupted testicular architecture, fibrotic remodeling, and seminiferous tubule vacuolation. Scale bar: 125 and 300 µm. C) According to structural disorganization in seminiferous tubules, fibrosis and vacuolar degeneration, the sections as in (B) were evaluated and scored for lesion severity using the following scoring system, which is scored as the sum of the three lesion scores: 0 = no lesions; 1 = minimal with lesions involving <5% of tissue; 2 = moderate with focally extensive areas of lesions (5–25% of tissue); 3 = moderate to severe with focally extensive areas of lesions (>25% to 50% of tissue); 4 = severe with large confluent areas of lesions (>50% of tissue). D) Transmission electron micrographs showing increased lysosomal abundance in infected testicular cells, red arrows indicate lysosomes. Scale bar: 2 µm. E) Statistical analysis of the lysosomes amounts of goat testis infected or not infected by *Brucella ssp*., as in (D). F) Immunofluorescence analysis of BLOC1S1 expression (red) in testicular tissue, with Hoechst 33342 counterstaining for nuclei (blue). Scale bar: 125 µm. G) Fluorescence intensity quantification of goat testis infected or not infected by *Brucella ssp*., as in (F). H) Relative *BLOC1S1* mRNA levels in goat testis infected or not infected by *Brucella ssp*. (*n* = 3). I) BLOC1S1 protein expression in o goat testis infected or not infected by *Brucella ssp*. J) Quantitative analysis of BLOC1S1 protein levels normalized to β‐actin (*n* = 3). ^*^ *p *< 0.05, ^**^ *p *< 0.01, ^***^ *p *< 0.001.

*Brucella* uses the host's regulated IRE1‐dependent decay (RIDD) pathway to impair the immune function, thus increasing its intracellular survival. The endonuclease activity of IRE1α, a critical ER stress sensor, is activated in *Brucella* infection to result in RIDD‐mediated degradation of select host mRNAs; notably among them, mitophagy receptor NIX and *Bloc1s1* mRNA encoding BLOC1S1, essential for lysosomal positioning events during endosome‐lysosome fusion. The activation of RIDD to cut out *Bloc1s1* mRNA and the inhibition on BORC complex, which is responsible for lysosomal trafficking by *Brucella*.^[^ ^24^, ^25^, ^26^, ^27^ ^]^ Venn analysis of RIDD‐associated genes and *Brucella*‐downregulated transcripts revealed significant overlap and identified *B* *loc1s1* and *P* *mp22* as the main candidate genes (**Figure** **1**A), which is consistent with previous studies.^[^ ^24^, ^25^, ^26^, ^27^ ^]^ BLOC1S1, a subunit of the BLOC‐1‐related complex (BORC), regulates lysosomal trafficking. Its degradation reduces lysosomal fusion with BCVs, thereby promoting intracellular bacterial proliferation.

Histopathological analysis of *Brucella*‐infected testicular tissues revealed disorganized seminiferous tubules, characterized by fibrosis, vacuolar degeneration, and loss of normal architecture (Figure 1B,C). Transmission electron microscopy (TEM) revealed a significant increase in lysosomal abundance within infected cells (Figure 1D,E). Immunofluorescence (IF) confirmed significant BLOC1S1 downregulation in infected seminiferous tubules (Figure 1F,G). Similarly, mRNA and protein detection of tissues also found that BLOC1S1 expression was significantly reduced (Figure 1H–J). These results demonstrate that *Brucella* infection disrupts testicular homeostasis and suppresses BLOC1S1 expression, mechanistically linking lysosomal dysregulation to enhanced bacterial survival.

### 2.2 BLOC1S1 Overexpression Elevates Lysosome‐Associated Gene Expression in mGSCs‐I‐SB

**Figure 2:**
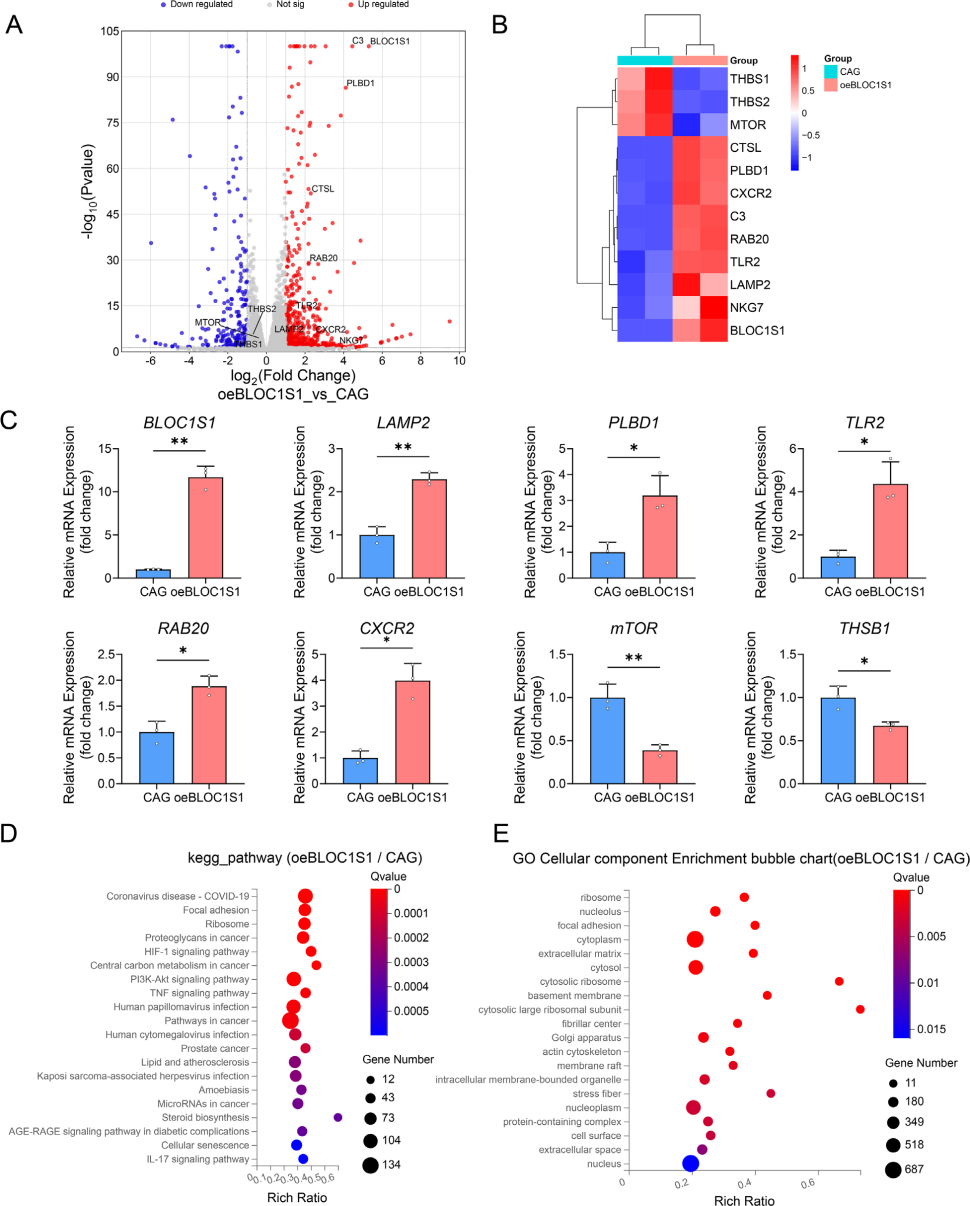
BLOC1S1 overexpression elevates lysosome‐associated gene expression in mGSCs‐I‐SB. A) Volcano plot displaying differentially expressed genes (DEGs) identified by RNA‐seq analysis between oeBLOC1S1 and CAG cells. B) RNA‐seq profiling of lysosome‐associated gene expression in oeBLOC1S1 versus CAG mGSCs‐I‐SB cells. C) Relative *BLOC1S1, LAMP2, PLBD1, TLR2, CXCR2, RAB20, mTOR* and *THBS1* mRNA levels in oeBLOC1S1 versus CAG mGSCs‐I‐SB cells (*n* = 3). D) Top 20 enriched KEGG pathways ranked by significance (*p*‐value) for upregulated and downregulated DEGs in oeBLOC1S1 versus CAG cells. E) Top 20 enriched GO biological processes by significance (*p*‐value) for upregulated and downregulated DEGs in oeBLOC1S1 versus CAG cells. ^*^ *p *< 0.05, ^**^ *p *< 0.01.

Based on the observed BLOC1S1 downregulation, we overexpressed BLOC1S1 (oeBLOC1S1) in mGSCs‐I‐SB (Figure S2A,B, Supporting Information). While BLOC1S1 overexpression did not induce morphological changes in mGSCs‐I‐SB, this was confirmed by a ninefold upregulation of mRNA expression (Figure S2C, Supporting Information) and a 1.6‐fold protein increase (Figure S2D,E, Supporting Information). RNA sequencing analysis of oeBLOC1S1 cells revealed upregulated of 1593 genes and downregulated of 1440 genes (**Figure** **2**A).

Focusing on lysosomal pathways consistent with the functional role of BLOC1S1, we identified significant upregulation of lysosome‐related genes (*CTSL*, *LAMP2*, *CXCR2*, *C3, RAB20, PLBD1*, and *NKG7*) alongside the expression of *mTOR* (a key regulator of TBF2 nuclear translocation) was downregulated (Figure 2B). The similar results were obtained using real‐time quantitative PCR (Figure 2C). KEGG pathway analysis highlighted enrichment in HIF‐1 signaling, PI3K‐AKT signaling, and TNF signaling pathways (Figure 2D). GO term analysis associated differential genes with ribosome, nucleolus, cytoplasm, and cytosol functions (Figure 2E). Collectively, these data demonstrate that BLOC1S1 overexpression drives transcriptional reprogramming toward lysosomal activation in mGSCs‐I‐SB cells.

### 2.3 *Brucella* 16M LPS Induces AMPK‐Dependent Autophagy in mGSCs‐I‐SB

**Figure 3:**
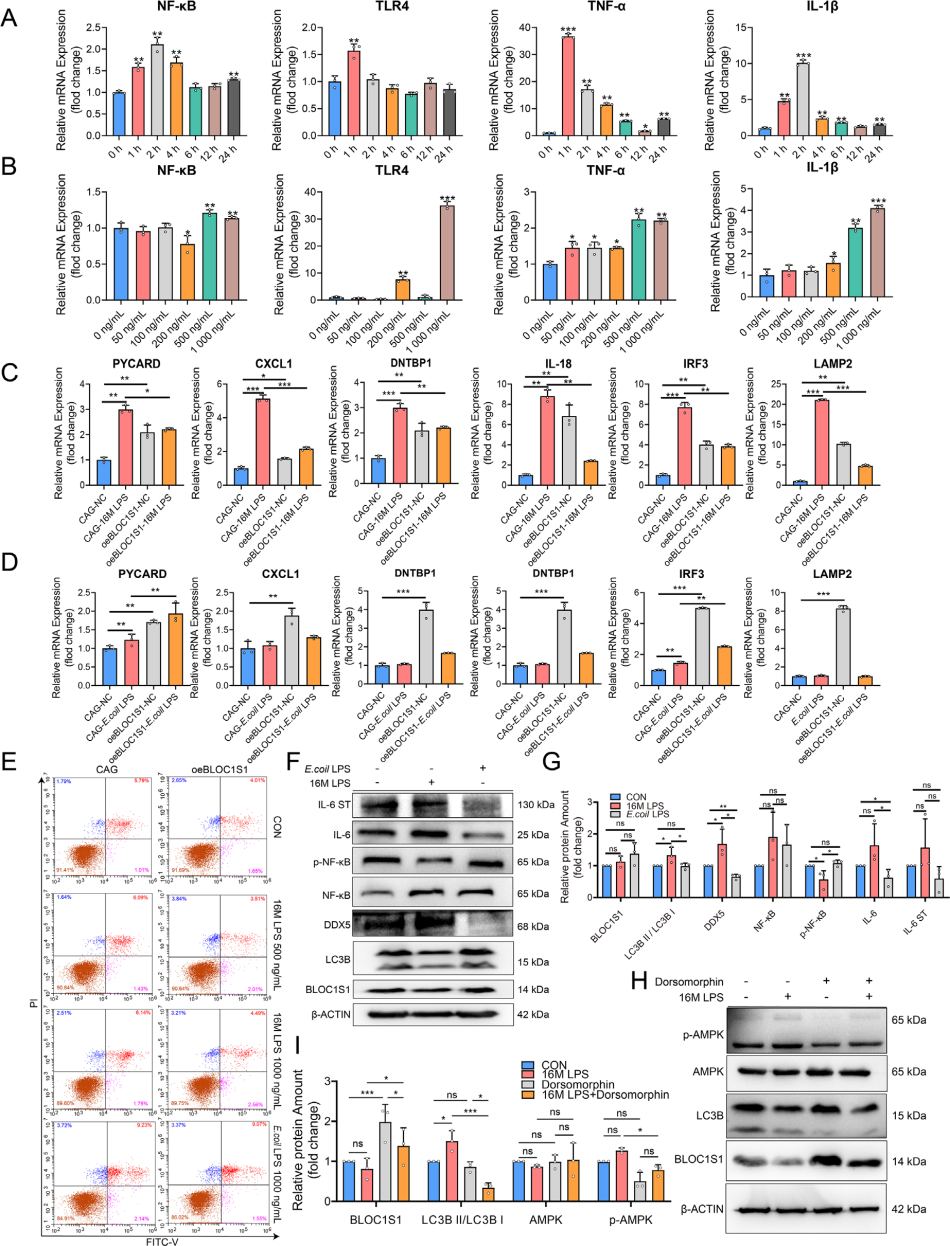
*Brucella* 16M LPS induces AMPK‐dependent autophagy in mGSCs‐I‐SB. A) Relative mRNA Levels of *NF‐κB*, *TLR4*, T*NF‐α*, and *IL‐1β* in mGSCs‐I‐SB cells (*n* = 3), these cells were treated with 16M LPS (500 ng/mL) for 0, 1, 2, 4, 6, 12, and 24 h. B) Relative mRNA Levels of *NF‐κB*, *TLR4*, T*NF‐α*, and *IL‐1β* in mGSCs‐I‐SB cells (*n* = 3), these cells were treated with 0, 50, 100, 200, 500, and 1000 ng/mL 16M LPS for 24 h. C) Relative mRNA Levels of *PYCARD*, *CXCL1*, *DNTBP1*, *IL‐18*, *IRF3*, and *LAMP2* in oeBLOC1S1 and CAG mGSCs‐I‐SB cells (*n* = 3), these cells were treated with 1000 ng/mL 16M LPS for 24 h. D) Relative mRNA Levels of *PYCARD*, *CXCL1*, *DNTBP1*, *IL‐18*, *IRF3*, and *LAMP2* in oeBLOC1S1 and CAG mGSCs‐I‐SB cells (*n* = 3), these cells were treated with 1000 ng/mL *E*.*coil* LPS for 24 h. E) Flow cytometry analysis of oeBLOC1S1 and CAG mGSCs‐I‐SB cells following staining with apoptosis‐specific probes Annexin V‐FITC/PI, these cells were treated with 500, 1000 ng/mL 16M LPS or 1000 ng/mL *E*.*coil* LPS for 24 h. F) The protein amounts of β‐ACTIN, BLOC1S1, LC3B, DDX5, NF‐κB, phospho‐NF‐κB, IL‐6, and IL‐6 ST in mGSCs‐I‐SB cells, these cells were treated with 1000 ng/mL 16M LPS or 1000 ng/mL *E*.*coil* LPS for 24 h. G) Statistical analysis of the protein amounts of BLOC1S1, LC3B, DDX5, NF‐κB, phospho‐NF‐κB, IL‐6, and IL‐6 ST in mGSCs‐I‐SB cells treated as in (F). The relative amounts of these proteins were normalized to β‐ACTIN. H) The protein amounts of β‐ACTIN, BLOC1S1, LC3B, AMPK, phospho‐AMPK in mGSCs‐I‐SB cells, these cells were treated with or without 16M LPS (1000 ng/mL) for 24 h in the presence or absence of AMPK inhibitor Dorsomorphin (10 µm). I) Statistical analysis of the protein amounts of BLOC1S1, LC3B, AMPK, phospho‐AMPK in mGSCs‐I‐SB cells treated as in (H). The relative amounts of these proteins were normalized to β‐ACTIN. ns: not significant. ^*^ *p *< 0.05, ^**^ *p *< 0.01, ^***^ *p *< 0.001.

To simulate *Brucella* infection, mGSCs‐I‐SB were treated with LPS isolated from 16M LPS. Initial time‐course experiments identified 1‐2 h as the peak period for inflammatory gene induction (**Figure** **3**A). However, based on prior evidence of BLOC1S1 downregulation at 24 h post‐infection,^[^ ^26^ ^]^ we selected 24 h for subsequent assays. Dose‐response analysis showed the highest expression of *TLR4*, *IL‐1β*, *TNF‐α* mRNA at 1000 ng/mL LPS (Figure 3B), which was determined to be the optimal concentration.

Comparative analysis with *E. coli* LPS showed that 16M LPS specifically upregulated proinflammatory (*PYCARD*, *CXCL1*, *IL‐18*, and *IRF3*) and lysosomal (*LAMP2*, *DNTPB1*) genes in control cells, with partial attenuation in oeBLOC1S1 cells (Figure 3C). In contrast, *E. coli* LPS showed no significant transcriptional activation (Figure 3D). Flow cytometry confirmed neither LPS type induced apoptosis in control or oeBLOC1S1 cells (Figure 3E; Figure S1F, Supporting Information).

Proteomic profiling revealed 16M LPS upregulated DDX5, NF‐κB, and IL‐6 while reducing phosphorylated NF‐κB (p‐NF‐κB) and IL‐6 ST. Conversely, *E. coli* LPS suppressed DDX5, IL‐6, and IL‐6 ST expression. The observed upregulation of *LAMP2* (Figure 3C) and an increase in the LC3B‐II/LC3B‐I ratio (Figure 3F,G) confirmed autophagy induction by 16M LPS. Mechanistic studies using the AMPK inhibitor dorsomorphin treatment abolished AMPK phosphorylation and autophagy activation (Figure 3H,I), confirming AMPK‐dependency. Crucially, AMPK inhibition reversed LPS‐induced BLOC1S1 downregulation (Figure 3H,I), establishing AMPK activation as the upstream driver of BLOC1S1 suppression. These results suggest that 16M LPS drives AMPK‐mediated autophagic flux in mGSCs‐I‐SB, while inversely modulating BLOC1S1 levels.

### 2.4 BLOC1S1 Overexpression Attenuates 16M LPS‐Induced Autophagic Activation

**Figure 4:**
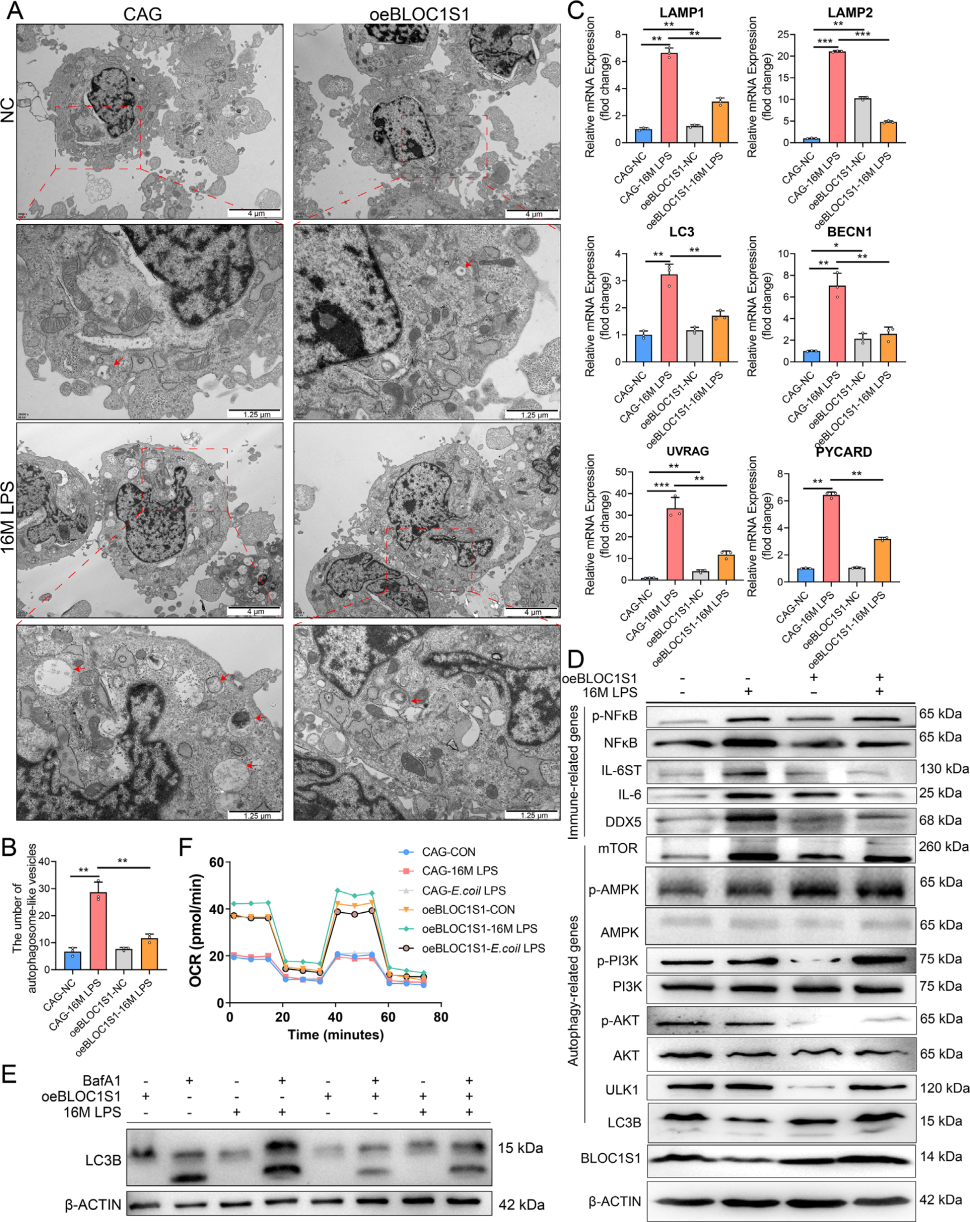
BLOC1S1 overexpression attenuates 16M LPS‐induced autophagic activation. A) Transmission electron microscopy visualization of cell junctions in oeBLOC1S1 or CAG mGSCs‐I‐SB cells treated with or without 16M LPS for 24 h. The red arrows indicate autophagosome‐like vesicles (autophagosomes and autolysosomes). The red box are higher magnification images. Scale bar: 2 and 0.5 µm. B) Statistical analysis of the autophagosome‐like vesicles amounts of in oeBLOC1S1 or CAG mGSCs‐I‐SB cells treated as in (A). C) Relative mRNA Levels of *LAMP1*, *LAMP2*, *LC3*, *BECN1*, *UVRAG*, and *PYCARD* in oeBLOC1S1 or CAG mGSCs‐I‐SB cells (*n* = 3), these cells were treated as (A). D) The protein amounts of β‐ACTIN, BLOC1S1, LC3B, ULK1, AKT, phospho‐AKT, PI3K, phospho‐PI3K, AMPK, phospho‐AMPK, mTOR, DDX5, NF‐κB, phospho‐NF‐κB, IL‐6, and IL‐6 ST in oeBLOC1S1 or CAG mGSCs‐I‐SB cells, these cells were treated as (A). E) The protein amounts of LC3B in oeBLOC1S1 or CAG mGSCs‐I‐SB cells, these cells were treated with or without 16M LPS (1000 ng/mL) for 24 h in the presence or absence of lysosomes inhibitor BafA1 (100 nM). F) Analysis of O_2_ consumption in oeBLOC1S1 or CAG mGSCs‐I‐SB cells treated with or without 16M LPS or *E*.coil LPS for 24 h. The rates of oxygen consumption (OCR) were first measured on 2×10^4^ cells of each groups under basal conditions and then with sequential additions of oligomycin (1.5 µm), FCCP (0.8 µm), rotenone (3.0 µm), and antimycin A (1.5 µm) at the indicated times to determine different parameters of mitochondrial functions. ^*^ *p *< 0.05, ^**^ *p *< 0.01, ^***^ *p *< 0.001.

To elucidate the regulatory role of BLOC1S1 in 16M LPS‐triggered autophagy, we performed ultrastructural, GFP‐mCherry‐LC3 tandem fluorescence analysis and molecular analyses. TEM confirmed that 16M LPS treatment significantly increased autophagosome‐like vesicles (autophagosomes and autolysosomes)^[^ ^28^ ^]^ formation in control (CAG) cells, while oeBLOC1S1 cells exhibited reduced autophagic vacuolization (**Figure** **4**A,B).

The tandem GFP‐mCherry‐LC3 construct serves as a reliable tool for monitoring autophagic flux. In this system: Yellow puncta (GFP^+^ mCherry^+^) indicate autophagosomes, red puncta (GFP^−^ mCherry^+^) represent autolysosomes. This distinction arises from GFP fluorescence quenching in the acidic lysosomal environment while mCherry remains stable.^[^ ^29^ ^]^ Consequently, transfection with the GFP‐mCherry‐LC3 plasmid in both CAG and oeBLOC1S1 cells revealed that CAG cells exhibited significantly increased red puncta (*p* < 0.001) and non‐significantly elevated yellow puncta under 16M LPS treatment, oeBLOC1S1 cells showed no increase in autolysosomes (red puncta) compared to untreated controls (Figure S2A,B, Supporting Information).

16M LPS upregulated autophagy‐related transcripts (*LAMP1*, *LAMP2*, *LC3*, *BECN1*, and *UVRAG*) in CAG cells, with significantly attenuated induction in oeBLOC1S1 cells (Figure 4C). Parallel protein level analysis showed that the LC3B‐II/LC3B‐I ratio in oeBLOC1S1 cells compared to 16M LPS‐treated controls (Figure 4D; Figure S2D, Supporting Information). Simultaneously, bafilomycin A1 (BafA1) was used to inhibit lysosomal acidification and thus suppress autophagic flux. CAG cells treated with 16M LPS exhibited elevated LC3B‐II/LC3B‐I ratio, while oeBLOC1S1 cells displayed a reduced ratio compared to CAG treated group (Figure 4E). Although statistical significance was not achieved due to substantial intra‐group variability (*p* > 0.05), the directional trend remained consistent across all experimental replicates (Figure S2C, Supporting Information).

Mechanistic analysis of upstream regulators showed that BLOC1S1 overexpression suppressed baseline levels of p‐PI3K, p‐AKT, and ULK1. While 16M LPS partially restored these phosphorylated signals, AMPK/p‐AMPK remained unaltered (Figure 4D; Figure S2D, Supporting Information). Importantly, LPS‐induced mTOR upregulation (potentially linked to oxidative stress responses) was attenuated in oeBLOC1S1 cells. These data suggest BLOC1S1‐mediated autophagy regulation operates independently of canonical PI3K/AKT‐mTOR‐ULK1 or AMPK‐ULK1 pathways. Concurrently, 16M LPS‐induced proinflammatory mediators (DDX5, IL‐6, IL‐6 ST, and NF‐κB/p‐NF‐κB) were attenuated in oeBLOC1S1 cells (Figure 4D; Figure S2D, Supporting Information). Metabolic analysis by oxygen consumption rate (OCR) assays further demonstrated enhanced mitochondrial function in oeBLOC1S1 cells, evidenced by elevated basal respiration, ATP‐linked respiration, maximal respiratory capacity, and spare respiratory capacity compared to controls (Figure 4F).

This study systematically explored the regulatory mechanism of BLOC1S1 overexpression on 16M LPS‐induced cellular responses through DIA proteomics. Principal component analysis (PCA) (Figure S3A, Supporting Information) showed that the CAG‐NC group and the CAG‐16M LPS group were clearly separated along the PC1 axis (contribution rate was 20.24%). oeBLOC1S1 cells (oeBLOC1S1‐NC and oeBLOC1S1‐16M LPS) displayed distinct separation from CAG groups in PCA space with high intra‐group reproducibility, suggesting BLOC1S1 may regulate inflammatory responses by stabilizing specific protein networks.

GO enrichment analysis (Figure S3D, Supporting Information) further revealed that BLOC1S1 overexpression significantly modulated biological processes including “nucleosome assembly” and “antigen processing/presentation,” along with molecular functions related to “cellular redox homeostasis,” implying its potential roles in epigenetic regulation and immune microenvironment remodeling. The enrichment of “nucleosome assembly” suggests BLOC1S1 may influence inflammatory gene expression through chromatin restructuring. KEGG pathway analysis uncovered treatment‐specific regulatory networks (Figure S3C,E,F, Supporting Information): In basal conditions (oeBLOC1S1‐NC vs CAG‐NC), “complement and coagulation cascades” showed marked activation (*p <0.001*), indicating BLOC1S1's potential to enhance innate immunity, while “cell adhesion molecules (CAMs)” enrichment supported its role in intercellular communication. The 16M LPS‐treated group (CAG‐16M LPS vs CAG‐NC) exhibited strong activation of “ferroptosis,” “lysosome,” and “cytokine‐cytokine receptor interaction” pathways (−log10(P) > 10), consistent with 16M LPS‐induced inflammatory damage and metabolic dysregulation. Following BLOC1S1 overexpression with LPS treatment (versus CAG‐16M LPS), “PPAR signaling” and “purine metabolism” pathways were significantly downregulated (Ratio<0.5), suggesting BLOC1S1 might attenuate 16M LPS proinflammatory effects by suppressing energy metabolism and lipid regulation. Significantly, BLOC1S1 overexpression specifically inhibited ferroptosis‐associated protein expression (Figure S3E, Supporting Information), a process intimately linked to inflammatory cell death, potentially representing a key anti‐inflammatory mechanism.

Collectively, these finding suggests that BLOC1S1 overexpression mitigates 16M LPS‐induced autophagic activation in mGSCs‐I‐SB through transcriptional and metabolic reprogramming.

### 2.5 BLOC1S1 Attenuates 16M LPS‐Induced Autophagy by Restricting TDP‐43 Nuclear Translocation

**Figure 5:**
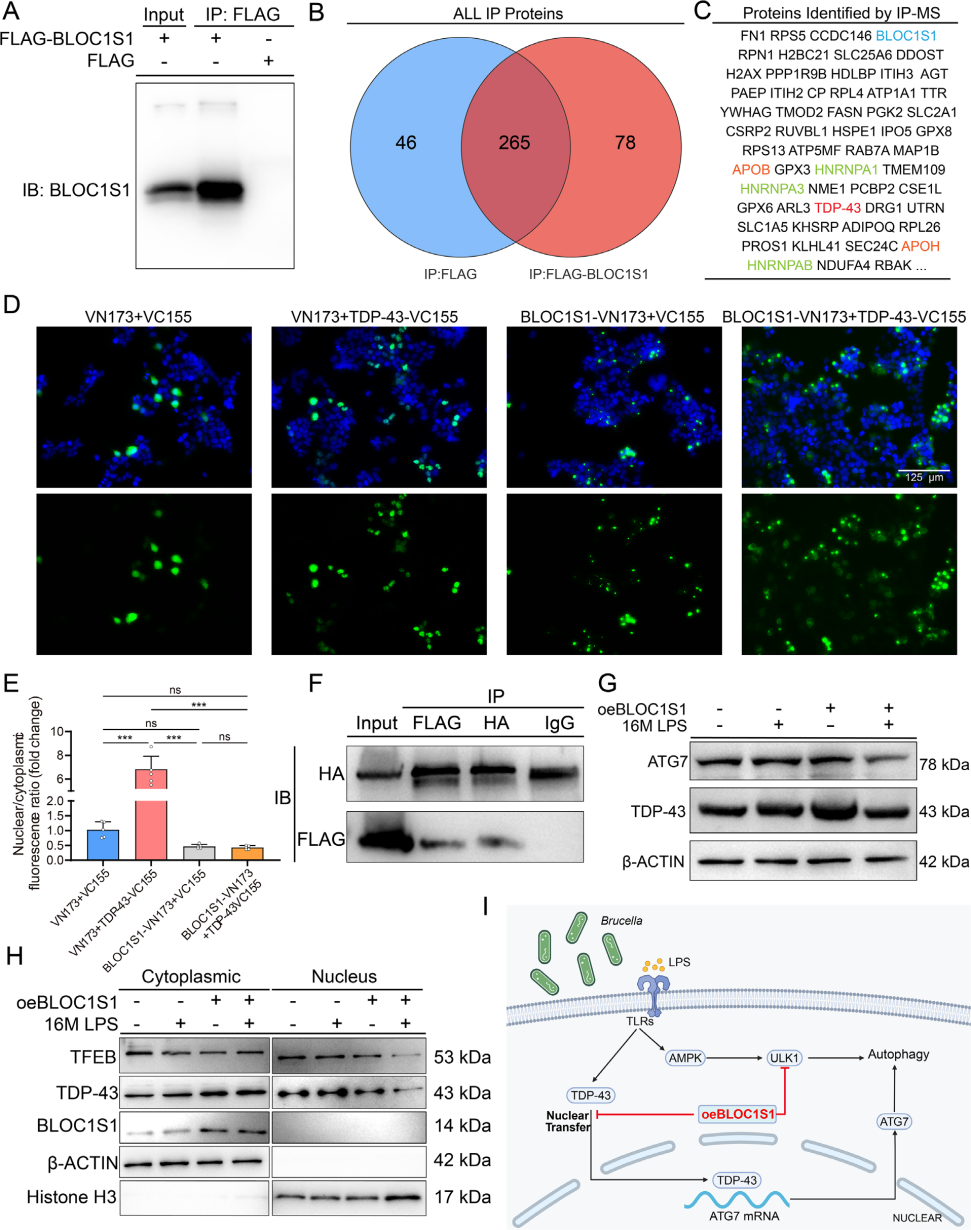
BLOC1S1 attenuates 16M LPS‐induced autophagy by restrictingTDP‐43 nuclear translocation. A) Total lysates were extracted from mGSCs‐I‐SB transfected by FLAG or FLAG‐BLOC1S1 fusion protein vectors for Co‐IP experiments. Equal number of proteins was immunoprecipitated separately with Anti‐FLAG M2 Magnetic Beads. Western blots show expression of BLOC1S1 with specific antibodies. B) Venn diagrams showing overlap of FLAG‐ and FLAG‐BLOC1S1‐associated proteins determined by mass spectrometry. C) Table lists FLAG‐BLOC1S1‐associated proteins. D) Two different fusion protein vectors, pBiFC‐VN173, pBiFC‐VC155, pBiFC‐VN173‐BLOC1S1, and pBiFC‐VC155‐TDP‐43 were transfected into HEK293T cells, and green fluorescence expression was observed after 48 h. Stronger green fluorescence indicates that the two proteins bind to each other. Cell nucleus were stained with DAPI (blue). Scale bar: 125 µm. E) Quantification of nuclear‐cytoplasmic ratio fluorescence signal for Figure 4D. F) Two different fusion protein vectors, pBiFC‐VN173‐BLOC1S1 and pBiFC‐VC155‐TDP‐43, were transfected into HEK293T cells, lysates were immunoprecipitated with anti‐FLAG or anti‐HA antibodies, and immunoprecipitated protein complex was examined. G) The protein amounts of β‐ACTIN, TDP‐43, ATG7 in oeBLOC1S1 or CAG mGSCs‐I‐SB cells, these cells were treated with or without 16M LPS for 24 h. H) The cytoplasmic and nuclear protein amounts of Histone H3, β‐ACTIN, BLOC1S1, TDP‐43, TFEB in oeBLOC1S1 or CAG mGSCs‐I‐SB cells, these cells were treated as (F). I) The 16M LPS induces elevated intracellular autophagy levels in mGSCs‐I‐SB cells through the AMPK pathway. BLOC1S1 interacts with TDP‐43, restricting its nuclear translocation, which subsequently reduces ATG7 expression and ultimately decreases autophagy levels (created by BioRender). ns: not significant, ^***^ *p *< 0.001.

To investigate the mechanism of BLOC1S1‐mediated autophagy suppression, we conducted co‐immunoprecipitation (Co‐IP) to identify BLOC1S1‐interacting proteins. Transfection of mGSCs‐I‐SB with FLAG‐BLOC1S1 or control plasmids, followed by immunoprecipitation and mass spectrometry, identified FLAG‐BLOC1S1‐associated proteins (**Figure** **5**A–C; Figure S2A, Supporting Information). These included lipid metabolism regulators: Apolipoprotein B (ApoB),^[^ ^30^ ^]^ Apolipoprotein H (ApoH),^[^ ^31^ ^]^ pre‐mRNA processing factors (hnRNPA1, hnRNPA3, hnRNPAB),^[^ ^32^, ^33^, ^34^ ^]^ and TDP‐43—a multifunctional RNA‐binding protein previously implicated in modulating autophagy.^[^ ^35^ ^]^ Specifically, TDP‐43 regulates the splicing of *raptor* and *Dynactin1* transcripts, which govern mTORC1‐mediated lysosomal biogenesis via TFEB and lysosome‐autophagosome fusion, respectively.^[^ ^18^ ^]^ In addition, TDP‐43 stabilizes ATG7, with its knockdown impairing autophagic flux.^[^ ^17^ ^]^ We therefore focused on TDP‐43 for mechanistic exploration.

AlphaFold 3 structural modeling predicted favorable BLOC1S1‐TDP‐43 binding interfaces (Figure S4B, Supporting Information).^[^ ^36^ ^]^ Bimolecular fluorescence complementation (BiFC) assays confirmed their interaction: Analogous to the positive control co‐transfection of pBiFC‐VN173‐BLOC1S1 and pBiFC‐VC155, co‐expression of pBiFC‐VN173‐BLOC1S1 and pBiFC‐VC155‐TDP‐43 demonstrated reconstituted GFP fluorescence specifically localized in the cytoplasm (Figure 5D,E). Co‐transfection of HEK293T cells with pBiFC‐VN173‐BLOC1S1 and pBiFC‐VC155‐TDP‐43 yielded FLAG‐ or HA‐tagged fusion proteins at predicted molecular weights (30 and 60 kDa; Figure 5F). Co‐immunoprecipitation (Co‐IP) further validated endogenous BLOC1S1‐TDP‐43 complexes in mGSCs‐I‐SB (Figure S4E, Supporting Information). Subcellular localization studies revealed nuclear TDP‐43 (punctate distribution) and cytoplasmic BLOC1S1; however, co‐transfection induced diffuse cytoplasmic co‐localization (Figure 4D; Figure S4C, Supporting Information).

Protein analysis showed that 16M LPS upregulated ATG7 and TDP‐43 levels in control (CAG) cells, whereas oeBLOC1S1 cells exhibited reduced ATG7 levels without changing TDP‐43 levels (Figure 5G; Figure S4H, Supporting Information). Immunofluorescence and nuclear‐cytoplasmic fractionation revealed LPS‐enhanced nuclear TDP‐43 accumulation in controls, which was abolished in oeBLOC1S1 cells (Figure 5H; Figure S4F,G,I, Supporting Information). This suggests BLOC1S1 restricts TDP‐43 nuclear import, diminishing its RNA splicing activity. Consequently, *ATG7* mRNA destabilization reduced ATG7 protein levels, suppressing 16M LPS‐triggered autophagy.

In summary, TLR‐mediated LPS recognition activates AMPK, promoting ULK1‐dependent autophagy initiation. Concurrently, cytoplasmic TDP‐43 translocates to the nucleus, stabilizing *ATG7* transcripts to amplify autophagic flux. BLOC1S1 counteracts both pathways by sequestering TDP‐43 in the cytoplasm (reducing ATG7 expression) and directly suppressing ULK1, thereby alleviating 16M LPS‐induced autophagy (Figure 5I).

## 3 Discussion

Our findings demonstrate that *B. melitensis* 16M‐derived LPS induces autophagy in mGSCs‐ISB, while BLOC1S1 attenuates this process by binding TDP‐43 to impede its nuclear translocation. This interaction inhibits TDP‐43‐dependent mRNA splicing, reducing ATG7 expression, and ultimately attenuates LPS‐induced autophagy.

Distinct from typical Gram‐negative bacterial LPS, *B. melitensis* 16M contains a core oligosaccharide with an O‐polysaccharide‐linked branch and a positively charged side chain. The latter may neutralize the negative charges of the core oligosaccharide, creating a “barrier” that prevents MD‐2/TLR4 receptor engagement and thus inhibits the immune responses.^[^ ^23^ ^]^ Consistent with previous observations, 16M LPS showed greater potency than *E. coli* LPS in inducing autophagy in goat spermatogonial stem cells.^[^ ^37^ ^]^ This property was further evidenced by 16M LPS‐driven upregulation of DDX5 and IL‐6, indicating enhanced host cell sensitivity.

OCR data (Figure 4F) demonstrate that BLOC1S1 overexpression enhances mitochondrial oxidative phosphorylation. This metabolic reprogramming likely contributes to autophagy suppression through energy‐sensing pathways: elevated ATP/NAD^+^ ratios inhibit AMPK while activating mTORC1, collectively repressing ULK1‐mediated autophagosome initiation (Figure 4D,E). These findings align with established metabolic control of autophagy,^[^ ^38^ ^]^ where mitochondrial enhancement restricts autophagic flux to conserve resources during stress. In *Brucella* infection, such BLOC1S1‐driven metabolic rewiring may starve bacteria of autophagic niches while exposing them to ROS.

BLOC1S1 participates in diverse cellular processes, including mitochondrial protein acetylation, metabolic regulation, and endosome‐lysosome trafficking.^[^ ^12^, ^13^ ^]^ As an acetyltransferase, it cooperates with acetyl‐CoA synthetases to maintain mitochondrial integrity via protein acetylation.^[^ ^39^ ^]^ It also recruits the BLOC1S1‐ΑTAT1 complex via RANBP2 to facilitate microtubule acetylation. Functionally linked to lysosomal dynamics, BLOC1S1 acts as a subunit of the BLOC‐1‐related complex (BORC), mediating lysosomal transport through ARL8 interaction.^[^ ^14^, ^15^ ^]^ Consistent with previous interactome studies, our IP‐MS analysis identified ARL8, ApoB, and ApoH as BLOC1S1 partners, underscoring its roles in lysosomal motility and lipid metabolism.^[^ ^16^, ^40^ ^]^ However, in addition to these established functions, our study reveals a novel role wherein BLOC1S1 spatially confines TDP‐43 in the cytoplasm, impeding its nuclear translocation. This sequestration disrupts TDP‐43‐dependent stabilization of *ATG7* mRNA,^[^ ^17^ ^]^ thereby attenuating 16M LPS‐triggered autophagy. This mechanism operates independently of canonical AMPK or PI3K/AKT‐mTOR pathways, instead relying on direct BLOC1S1‐TDP‐43 interaction.

During *Brucella* infection, the T4SS effector VceC induces the unfolded protein response (UPR)‐regulated IRE1α‐dependent decay (RIDD) pathway, degrading *Bloc1s1* mRNA and disrupting BORC assembly.^[^ ^41^ ^]^ Reconstitution of BLOC1S1 via *Bloc1s1* G360C mutations, which preserve protein function while evading IRE1αmediated degradation, restores lysosomal trafficking and reduces rBCV formation.^[^ ^16^, ^26^ ^]^ Our study expands BLOC1S1's functional repertoire by revealing its ability to sequester TDP‐43 in the cytoplasm, thereby limiting nuclear TDP‐43 activity.^[^ ^17^ ^]^ TDP‐43 loss or cytoplasmic mislocalization is implicated in aberrant lysosomal biogenesis, autophagosome‐lysosome fusion defects,^[^ ^18^ ^]^ and neurodegenerative disorders like amyotrophic lateral sclerosis (ALS) and frontotemporal dementia (FTD).^[^ ^19^, ^20^ ^]^ Critically, TDP‐43 mislocalization is a hallmark of ALS and FTD, where cytoplasmic aggregation drives neurodegeneration via loss of nuclear RNA‐processing functions.^[^ ^42^ ^]^ Notably, TDP‐43 knockout elevates cryptic exon inclusion, a phenotype reversible upon TDP‐43 reintroduction or antisense oligonucleotide treatment.^[^ ^43^, ^44^, ^45^ ^]^ Our finding that BLOC1S1 retains TDP‐43 in the cytoplasm—thereby mimicking the pathological state in ALS/FTD yet conferring protection against bacterial persistence—suggests a dual‐pathogenenic role for TDP‐43 dysregulation. Given BLOC1S1's capacity to modulate TDP‐43 nucleocytoplasmic shuttling, it warrants investigation as a potential therapeutic target for TDP‐43 proteinopathies. RIDD‐mediated degradation of BLOC1S1 mRNA can promote the degradation of Huntington's protein, thereby alleviating the progression of Huntington's disease.^[^ ^46^ ^]^ Therefore, restoring TDP‐43 nuclear function rescues autolysosome deficits in neurodegeneration, BLOC1S1's ability to modulate TDP‐43 shuttling positions it as a potential therapeutic node for both bacterial infections and TDP‐43‐mediated neuropathologies.

The BLOC1S1‐TDP‐43‐ATG7 autophagy regulatory axis demonstrates profound evolutionary conservation across goats, mice, and humans. At the sequence level, BLOC1S1 maintains >98% homology among these species and universally serves as a core subunit of the BORC complex, mediating lysosomal trafficking through the ARL8 GTPase.^[^ ^16^ ^]^ TDP‐43 exhibits equally high conservation, particularly in its RNA recognition motifs (RRMs) and nuclear localization signal (NLS), with demonstrated roles in stabilizing *ATG7* mRNA across mammalian models—including mouse neurons, Hela cells, and caprine spermatogonial cells.^[^ ^17^, ^18^, ^47^ ^]^ The high conservation of their protein sequences provides a molecular basis for the preserved BLOC1S1‐TDP‐43 interaction across species, thereby confirming the functional conservation of the BLOC1S1‐TDP‐43‐ATG7 autophagy axis. In studies of *Brucella*‐infected macrophages, the pathogen degrades BLOC1S1 via the RIDD pathway, disrupting BORC complex assembly. In *Brucella*‐infected macrophages, pathogen‐mediated RIDD activation degrades BLOC1S1, disrupting BORC complex assembly.^[^ ^26^ ^]^ As shown in our study, BLOC1S1 depletion enhances nuclear translocation of TDP‐43, thereby stabilizing ATG7 expression. Although ATG7 is dispensable for aBCV biogenesis, a critical compartment for *Brucella* replication, paradoxically, ATG7 knockdown increases aBCV formation.^[^ ^10^ ^]^ Thus, the BLOC1S1‐TDP‐43‐ATG7 axis functions to suppress aBCV generation during macrophage infection, representing a host‐protective mechanism that limits bacterial dissemination.

Future studies will elucidate the structural basis of BLOC1S1‐TDP‐43 interactions through cryo‐EM and mutagenesis to identify druggable interfaces, while validating this axis in primary macrophages and iPSC‐derived motor neurons to assess its conservation across cellular contexts. Therapeutic strategies will be developed to engineer bifunctional compounds that either enhance BLOC1S1‐TDP‐43 binding to combat intracellular pathogens like Brucella or disrupt this interaction to restore nuclear TDP‐43 function in ALS/FTD models. Parallel clinical investigations will correlate BLOC1S1 polymorphisms with disease progression in brucellosis and TDP‐43 proteinopathy cohorts, bridging fundamental mechanisms to precision medicine applications across infectious and neurodegenerative diseases.

This study elucidates a previously unrecognized mechanism whereby BLOC1S1 restricts LPS‐driven autophagy through spatial regulation of TDP‐43, complementing its canonical roles in organelle trafficking and post‐translational modification.

## 4 Experimental Section

### Collection of Goat Testicular Tissue

All procedures involving experiments with Brucella‐infected testicular tissues were reviewed and approved by the Bioethics and Biosafety Management Committee of Yulin University (Approval No. 2022051306). Testicular tissue was obtained from a 6‐year‐old goat naturally infected with *Brucella*. The sample was sourced from the Yulin Animal Husbandry and Veterinary Service Center. Healthy control samples (6‐year‐old) were selected from Brucella‐free farms in the same region and confirmed negative through the Rose Bengal plate test (RBT).^[^ ^48^ ^]^

### Vector Construction

All primers used in this study are listed in **Table** **1**. Following target gene amplification, homologous arms matching the restriction enzyme‐digested vector termini were added to PCR products using the NovoRec Plus One‐Step PCR Cloning Kit (Novo, China) for homologous recombination. Some plasmids were constructed using T4 ligation (T4 DNA Ligase, Thermo Scientific, USA), such as: CMV‐FLAG‐BLOC1S1‐mCherry and CMV‐HA‐TDP‐43‐EGFP. Plasmids extracted from DH5α cells were sequenced, and sequence‐verified constructs were selected for subsequent experiments. The following expression vectors were generated: CAGG‐BLOC1S1‐Puro: for BLOC1S1 overexpression, pBiFC‐VN173‐BLOC1S1, pBiFC‐VC155‐TDP‐43, EF1‐3×FLAG‐BLOC1S1, CMV‐FLAG‐BLOC1S1‐mCherry, CMV‐HA‐TDP‐43‐EGFP, and GFP‐mCherry‐LC3 for transfected cells.

**Table 1:** Forward and reverse primers for vector construction.

Primer	Primer sequence (5′→3′)	Size (bp)
CAGG‐*BLOC1S1*‐F	agctgcagatatcGAATTCGATGCTGTCCCGCTTGCTGAA	40
CAGG‐*BLOC1S1*‐R	ttcaccggcatctGGATCCGCTAGGAGGGGGCCGACTGCA	40
EF1‐3×FLAG‐*BLOC1S1*‐F	atgacgatgacaagGAATTCATGCTGTCCCGCTTGCTGAA	40
EF1‐3×FLAG‐*BLOC1S1*‐R	cttcctctgccctcGGATCCGGAGGGGGCCGACTGCAGCT	40
CMV‐FLAG‐*BLOC1S1*‐mCherry‐F	gGAATTCgccaccatgGACTACAAAGACGATGACGACAAGATGCTGTCCCGCTTGCTGAA	59
CMV‐FLAG‐*BLOC1S1*‐mCherry ‐R	gGGTACCaaGGAGGGGGCCGACTGCAGCT	28
CMV‐HA‐*TDP‐43*‐EGFP‐F	cAAGCTTcgccaccatgTACCCATACGATGTTCCAGATTACGCTATGTCTGAATATATTCGGGT	64
CMV‐HA‐*TDP‐43*‐EGFP‐R	cGGATCCaaCATTCCCCAGCCAGAAGACT	28
pBiFC‐VN173‐*BLOC1S1*‐F	aagcttgcggccgcgAATTCcATGCTGTCCCGCTTGCTGAA	41
pBiFC‐VN173‐*BLOC1S1*‐R	agtcgactggtaccGATATCagGGAGGGGGCCGACTGCAGCT	42
pBiFC‐VC155‐*TDP‐43*‐F	ggccatggaggcccGAATTCgtATGTCTGAATATATTCGGGT	42
pBiFC‐VC155‐*TDP‐43*‐R	ctcgagagatctcgGTCGACacCATTCCCCAGCCAGAAGACT	42

F: Forward primer; R: Reverse primer. The bold sequence indicates an enzyme cleavage site, and the underlined lowercase letter sequence indicates a homologous arm sequence. Underlined capital letters indicate FLAG or HA labels. The remaining lowercase letters indicate a protective base, a Kozak sequence, or a base that prevents frameshift mutations.

### Cell Culture and Transfection

HEK293T cells were maintained in Dulbecco's Modified Eagle Medium (DMEM; Corning, USA) supplemented with 10% heat‐inactivated fetal bovine serum (FBS; Gibco, USA), penicillin (100 IU/mL), and streptomycin (100 IU/mL; Thermo Fisher Scientific, USA) at 37 °C under 5% CO_2_. mGSCs‐I‐SB were cultured in DMEM/F12 (Corning, USA) containing 5% heat‐inactivated horse serum, 2.5% heat‐inactivated FBS, penicillin (100 IU/mL), and streptomycin (100 IU/mL) under identical incubation conditions.^[^ ^49^ ^]^ Cell transfection was performed using jetPRIME transfection reagent (Polyplus, USA) following the manufacturer's protocol. All cells were obtained from the Laboratory Animal Center of Northwest A&F University. The mGSCs‐I‐SB cell line was originally isolated, cultured, established, and cryopreserved by this research group.

### Construction of Stably Transfected Cell Lines

Lentiviral supernatant preparation followed established protocols from the previous study.^[^ ^50^ ^]^ Briefly, 293T cells were thawed, expanded to ≈80% confluence in optimal condition, and transfected with 8 µg total plasmid DNA (target plasmid:PAX2:VSVG = 4:3:2 ratio) using jetPRIME. Complete medium replacement was performed 12 h post‐transfection, with viral supernatant harvested after 48 h incubation without disturbance.

Collected lentiviral supernatant was centrifuged at 2000 rpm for 5 min, filtered through 0.45 µm membranes, and supplemented with Polybrene (1:500 dilution) before transduction. mGSCs‐I‐SB cells were infected with a 1:1 mixture of lentivirus and DMEM/F12 complete medium. After 12 h exposure, cells reverted to standard culture conditions. Puromycin selection (10 µg/mL) commenced 72 h post‐infection.

Negative control mGSCs‐I‐SB cells (untreated) showed complete mortality within 72 h under identical puromycin conditions. Medium containing consistent puromycin concentrations was refreshed every 24 h during selection. Stable transfected clones were isolated following three days of continuous selection after full clearance of control cells.

### LPS Treatment

Cells were treated with *E. coli*‐derived LPS (Sigma L2630, USA) or *Brucella melitensis* 16M strain‐derived LPS, with equal‐volume PBS serving as negative controls. Cells were harvested at designated time points for downstream analyses. Cells were harvested at 0 h, 1 h, 2 h, 4 h, 6 h, 12 h or 24 h for downstream analyses.

### RNA Extraction, Reverse Transcription, and Quantitative Real‐Time PCR

qRT‐PCR analysis was performed following established methodology.^[^ ^51^ ^]^ Total RNA was extracted from cells using TRIzol Reagent (TaKaRa, #9108, Japan). First‐strand cDNA synthesis was conducted with a reverse transcription kit (Thermo Fisher Scientific, USA). qRT‐PCR amplification was performed on a CFX96 Real‐Time System (Bio‐Rad, USA) using SYBR Green Premix (Vazyme Biotech, #Q311‐02, China). Gene‐specific primer sequences are provided in **Table** **2**.

**Table 2:** The primer for qRT‐PCR.

Primer	Primer sequence (5′→3′)	Size (bp)
Q‐BLOC1S1‐F	AGGAAGCTTGACCATGAGGTG	21
Q‐BLOC1S1‐R	TCCTTGAGTGCCTGGTTGAAG	21
Q‐NF‐κB‐F	TGGGGATACTGAACAACGCC	20
Q‐NF‐κB‐R	ATCTGTCTCAGGGCCTCCAT	20
Q‐IRF3‐F	AACCTGGGGCCCTTCATAAC	20
Q‐IRF3‐R	CAACCTTGACCATCACAAGCC	21
Q‐CXCL1‐F	TCAAGACTGGTCAGGAAGTGTG	22
Q‐CXCL1‐R	TGGAGCTGGCCTGGTTTAG	22
Q‐JUN‐F	ACGACCTTCTACGACGATGC	21
Q‐JUN‐R	GCCAGATTCAGGGTCATGCT	21
Q‐β‐ACTIN‐F	TGATATTGCTGCGCTCGT	18
Q‐β‐ACTIN‐R	CTTGAGGGTCAGGATGCC	19
Q‐ASC‐F	GCCGTGGACCTTACCGACAA	20
Q‐ASC‐R	GCAGTCCTGGCTTGGCTATCTT	22
Q‐TLR4‐F	GGGTGCGGAATGAACTGGTA	20
Q‐TLR4‐R	CTGGGACACCACGACAATCA	20
Q‐LAMP2‐F	GCAACAAAGAGCAGACCGTT	20
Q‐LAMP2‐R	TGGGCACAAGGAAGTTGTCG	20
Q‐DTNBP1‐F	GAAGATACATGGGCTGCGCT	20
Q‐DTNBP1‐R	TTGCCGTCAGGGACTCTAAG	20
Q‐IL‐18‐F	GCCAGGGAAATCAACCTGTCT	21
Q‐IL‐18‐R	GACCTCTAGTGAGGCTGTCCT	21
Q‐LAMP1‐F	ACTTTCGGAAGAGGCCACAC	20
Q‐LAMP1‐R	AGTTGCTGCTGGACAGGTAG	20
Q‐BECN1‐F	CTCTCGCAGATTCATCCCCC	20
Q‐BECN1‐R	GTCTTCGGCTGAGGTTCTCC	20
Q‐UVRAG‐F	GTCTCAGCAGCGTCGTCTTA	20
Q‐UVRAG‐R	GATGTCCTCCTTTCCACCCC	20
Q‐LC3‐F	TTTGCCGACCGCTGTAAAGA	20
Q‐LC3‐R	CCCTTGTAGCGCTCGATGAT	20
Q‐IL‐1β‐F	GAAGAGCTGCACCCAACA	18
Q‐IL‐1β‐R	CAGGTCATCATCACGGAAG	19
Q‐TNF‐α‐F	CCGGTAGCCCACGTTGTAG	19
Q‐TNF‐α‐R	GCGAGTAGATGAGGTAAAGCCC	22
Q‐CXCR2‐F	AGCGGTATTCTACTGCTGGC	20
Q‐CXCR2‐R	ACTGGAATAGGGTGGGTGGA	20
Q‐PLBD1‐F	TGGGACATGGGACATTGCTC	20
Q‐PLBD1‐R	ACTGCTGAAAGAGAGGCGAC	20
Q‐RAB20‐F	CGGGAAGATCGTGCTTTTGG	20
Q‐RAB20‐R	GAGATGTTGAAGGAGCGCCA	20
Q‐TLR2‐F	TGGGCTGTAATCAGCGTGTT	20
Q‐TLR2‐R	TCGTTGTCGGACAGGTCAAG	20
Q‐MTOR‐F	CTATCCGTGTGTTGGGGCTT	20
Q‐MTOR‐R	TGAGAGAGGGACTGGTCTCG	20
Q‐THBS1‐F	AGCCTCAACAACAGATGCGA	20
Q‐THBS1‐R	CCCTCACATGGCTTCCCATT	20

F: Forward primer; R: Reverse primer; Q: Primer sequence for qRT‐PCR.

### Western Blotting, Immunoblotting, and Co‐Immunoprecipitation

Western blotting performed as previous described^[^ ^52^ ^]^ using antibodies detailed in **Table** **3**. Cells were lysed in NP‐40 buffer (Beyotime, China) containing phosphatase and protease inhibitors. Lysates were centrifuged at 2000 rpm for 20 min at 4 °C to collect proteins. Protein concentrations were quantified via Bradford assay (Tiangen Biotech, #PA102, China), denatured in loading buffer, and separated via sodium dodecyl sulfate–polyacrylamide gel electrophoresis (SDS‐PAGE). Signals were detected using a Bio‐Rad imaging system (USA) and quantified with ImageJ. Statistical significance was determined by Student's *t*‐test. Nuclear and cytoplasmic proteins were extracted using a fractionation kit (Beyotime, #P0027, China) per manufacturer instructions. Full uncropped Western blot images are provided in the Supporting Information file.

**Table 3:** Antibodies used in this study.

Antibody	Supplier	Cat.No.
NF‐κB	Abmart	T55034F
p‐NF‐κB	Abmart	TP56372F
IL‐6	Wanleibio	WL02841
IL‐6‐ST	Proteintech	67766‐1‐Ig
DDX5	ImmunoWay	YT3548
LC3B	Abmart	T55992F
BLOC1S1	Santa Cruz	SC‐515444
β‐ACTIN	ABclonal	AC038
mTOR	ImmunoWay	TY2913
ULK1	Abmart	T56902F
PI3K	ImmunoWay	YM3503
p‐PI3K	ImmunoWay	YP0224
AKT1	ImmunoWay	YT0185
p‐AKT1	ImmunoWay	YP0006
AMPK	Abmart	T55326F
p‐AMPK	Abmart	TA3423F
TDP‐43	ImmunoWay	PT0126R
TFEB	Proteintech	13372‐1‐AP
ATG7	Proteintech	10088‐2‐AP
FLAG	Sigma	F1804
Goat Anti‐Mouse IgG (H+L)	BOSTER	BA1050
Goat Anti‐Rabbit IgG (H+L)	BOSTER	BA1054

For IP, mGSCs‐I‐SB cells were transfected by EF1‐3×FLAG‐BLOC1S1 and EF1‐3×FLAG‐empty plasmids. For Co‐IP, HEK293T cells were transfected by two of the four plasmids, i,e, pBiFC‐VN173‐BLOC1S1, pBiFC‐VC155‐TDP‐43, pBiFC‐VN173‐empty, pBiFC‐VC155‐empty. After transfection for 48 h, cells were lysed in NP‐40 buffer (Beyotime, #P0013, China) for 30 min. Lysates were centrifuged at 12000 rpm for 10 min at 4 °C, and supernatants were incubated with anti‐FLAG M2 magnetic beads (Millipore, USA) or anti‐HA beads (Beyotime, #P2121, China) for 4 h at 4 °C. Beads were washed five times and eluted in 1× SDS‐PAGE loading buffer. Samples were boiled, resolved by SDS‐PAGE, and transferred to polyvinylidene fluoride (PVDF) membranes. Immunoblotting was performed with specified antibodies.^[^ ^50^ ^]^ Coomassie blue staining (Tiangen Biotech, #PA101, China) was conducted per manufacturer protocols.

### AMPK Inhibition

To investigate the effects of AMPK inhibition in mGSCs‐I‐SB, cells were divided into four experimental groups: control, Dorsomorphin‐treated (10 µm; HY‐13418A, MedChemExpress),^[^ ^53^, ^54^ ^]^ 16M LPS‐treated (1000 ng/mL), and Dorsomorphin/16M LPS co‐treated groups. The ATP‐competitive AMPK inhibitor Dorsomorphin and 16M LPS were administered for 24 h. Protein lysates were subsequently collected for Western blotting analysis following the treatment period.

### Autophagic Flux Assay

Cells were transfected with GFP‐mCherry‐LC3 vector for 48 h and then treated with or without 16M LPS (1000 ng/mL) for 24 h. Cells were fixed with 4% paraformaldehyde (Sigma, USA) for 15 min and washed three times with PBS for 5 min each. Nuclear staining was performed with DAPI. After staining, the cells were washed three more times with PBS for 5 min each. Recording using a fluorescence microscope (AMG, USA). The The yellow puncta represent autophagosomes (GFP positive and mCherry positive) and red puncta represent autolysosomes (GFP negative and mCherry positive).

### Transmission Electron Microscope Assay

Prefixed with a 3% glutaraldehyde, then the tissue was postfixed in 1% osmium tetroxide, dehydrated in series acetone, infiltrated in Epox 812 for a longer, and embeded. The semithin sections were stained with methylene blue and Ultrathin sections were cut with diamond knife, stained with uranyl acetate and lead citrate. Sections were examined with JEM‐1400‐FLASH Transmission Electron Microscope.

### Bafilomycin A1 Treatment

To investigate the LC3B‐II/LC3B‐I, CAG cells and oeBLOC1S1 cells were divided into four experimental groups: control, BafA1‐treated (100 nM; HY‐100558, MedChemExpress), 16M LPS‐treated (1000 ng/mL), and BafA1/16M LPS co‐treated groups. The cells were treated for 24 h. Protein lysates were subsequently collected for Western blotting analysis following the treatment period.

### Oxygen Consumption Rate Assay

Seed mGSCs‐I‐SB cells (CAG or oeBLOC1S1) in XF96 cell culture microplates (Agilent, #102416‐100) at 2.0×10⁴ cells/well in 80 µL complete DMEM/F12 medium. Treat cells with 1000 ng/mL 16M LPS, *E. coli* LPS, or PBS control for 24 h. Hydrate XFe96 Sensor Cartridge (Agilent, #102416‐100) in 200 µL well^−1^ XF Calibrant (Agilent, #100840‐000) at 37 °C (no CO_2_) for 24 h. Replace medium with 180 µL/well assay medium: DMEM (Agilent, #103575‐100) with 10 mM glucose, 1 mM pyruvate, 2 mM glutamine (pH 7.4). Equilibrate at 37 °C (no CO_2_) for 1 h. Load compounds into injection ports: Port A: 1.5 µm oligomycin (in assay medium). Port B: 0.8 µm FCCP (carbonyl cyanide‐4‐(trifluoromethoxy) phenylhydrazone). Port C: 3.0 µm rotenone with 1.5 µm antimycin A. Run assay on Seahorse XFe96 Analyzer (Agilent) using standard cycle.

### Immunofluorescence Staining

Cells were seeded onto glass coverslips in 48‐well plates and treated with 16M LPS for 24 h. Cells were fixed with 4% paraformaldehyde (PFA) at room temperature for 15 min, followed by three PBS washes (5 min each) on a shaker. Fixed cells were blocked with 10% fetal bovine serum (FBS) at 4 °C for 1 h to reduce nonspecific binding. The blocking solution was removed, and cells were incubated with primary antibodies diluted 1:100 in 10% FBS at 4 °C overnight. After removing the primary antibodies, cells were washed three times with PBS (5 min each). Fluorescent secondary antibodies (1:300 dilution in 10% FBS) were applied and incubated at 37 °C for 1 h under light‐protected conditions. Following secondary antibody removal and PBS washes, cells were counterstained with 100 µL of DAPI‐containing antifade mounting medium to visualize nuclei. Fluorescent images were captured using a fully automated imaging system (Nikon, Eclipse Ti2‐E). Antibody details are listed in Table 3.

### Bimolecular Fluorescence Complementarity

Two different fusion protein vectors, pBiFC‐VN173, pBiFC‐VC155, pBiFC‐VN173‐BLOC1S1 and pBiFC‐VC155‐TDP‐43, were transfected into HEK293T cells, and green fluorescence expression was observed after 48 h. Cells were fixed with 4% paraformaldehyde (Sigma, USA) for 15 min and washed three times with PBS for 5 min each. Nuclear staining was performed with DAPI. After staining, the cells were washed three more times with PBS for 5 min each. The green fluorescent protein (GFP) of cells was observed and recorded using a fluorescence microscope (AMG, USA). Stronger green fluorescence indicates that the two proteins bind to each other.

### Protein Colocalization Analysis

HEK293T cells were co‐transfected with CMV‐FLAG‐BLOC1S1‐mCherry and CMV‐HA‐TDP‐43‐EGFP constructs. Post‐transfection, cells were fixed, stained with DAPI, and mounted on glass slides. Colocalization was visualized using a laser scanning confocal microscope (Leica TCS SP8 SR; Leica Microsystems, Germany). Images were acquired and analyzed using LAS X software (Leica) to assess spatial protein interactions.

### RNA Sequencing and Data Analysis

To investigate BLOC1S1‐mediated molecular regulation in vitro, transcriptome sequencing was performed on mGSCs‐I‐SB cells (CAG and oeBLOC1S1 groups) with two biological replicates through BGI (China). Total RNA was extracted using TRIzol Reagent (TaKaRa, #9108, Japan) following the manufacturer's protocol. RNA quantity was assessed by spectrophotometry (NanoDrop One, Thermo Fisher Scientific, USA), and quality was verified via 1.5% agarose gel electrophoresis. DNA contamination was removed using DNase I, followed by RNA quantification with a Qubit RNA Broad‐Range Assay Kit (Life Technologies, #Q10210, USA) on a Qubit v3.0 fluorometer.

Stranded RNA‐seq libraries were prepared using the KC‐Digital Stranded mRNA Library Prep Kit for Illumina (DR08502, Seqhealth, China) with 2 µg total RNA. This system incorporates unique molecular identifiers (UMIs) containing 8 random bases to eliminate PCR/sequencing duplication biases. Library fragments (200–500 bp) were enriched, quantified, and sequenced on a NovaSeq 6000 platform (Illumina, USA) using PE150 mode.

Raw sequencing reads were processed with Trimmomatic (v0.36) to remove adapters and low‐quality bases. UMI‐based deduplication was performed using in‐house scripts: Clean reads were clustered by UMI sequences, and reads within clusters were aligned pairwise. Subclusters with >95% sequence identity were consolidated into consensus sequences via multiple sequence alignment, effectively removing PCR/sequencing artifacts.

Deduplicated consensus reads were aligned to the goat reference genome (*Capra hircus*, assembly ARS1; NCBI accession) using STAR (v2.5.3a) with default parameters. FeatureCounts (Subread‐1.5.1) quantified exonic reads, normalized as reads per kilobase per million (RPKM). Differential expression analysis was conducted using edgeR (v3.12.1), with significance thresholds set at |log2(fold change)| ≥1 and adjusted *p* < 0.05. Functional enrichment analysis (GO and KEGG pathways) was performed via KOBAS (v2.1.1), retaining terms with *p* < 0.05 after multiple testing correction.

### DIA Proteomic Sequencing and Data Analysis

Protein samples from 16M LPS‐treated or untreated CAG/oeBLOC1S1 cells (two biological replicates per group) were subjected to data‐independent acquisition (DIA) mass spectrometry at Novogene Co., Ltd (China). MS analysis was performed on an anquish neo‐astral platform. Raw files were processed using DIA‐NN (v1.8.1) with the following parameters: Global precursor q‐value < 0.01 and protein group q‐value < 0.01. The UniProt *Capra hircus* reference proteome database (release 2023_10_18; 77735 sequences) was employed for peptide identification. Differentially expressed proteins (DEPs) were defined as those showing >1.5‐fold change with *p* < 0.05 by Student's *t*‐test. Functional annotation and pathway enrichment analyses were subsequently conducted using STRING (v12.0) and Metascape platforms.

### Liquid Chromatography‐Tandem Mass Spectrometry

To identify BLOC1S1‐interacting proteins, two independent replicates of 3×FLAG control and 3×FLAG‐BLOC1S1 samples were prepared. Immunoprecipitation (IP) procedures followed the aforementioned Co‐IP protocol. Protein complexes were resolved via 10% SDS‐PAGE, and excised gel bands underwent in‐gel tryptic digestion. Processed peptides were analyzed by LC‐MS/MS using an Orbitrap Fusion Lumos Tribrid Mass Spectrometer (Thermo Fisher Scientific, USA) at Jingjie PTM BioLabs (China).

MS/MS data were processed through Proteome Discoverer v1.3 against the UniProt database (release 2023_10).^[^ ^55^ ^]^ Proteins immunoprecipitated in the 3×FLAG control group served as background signals and were subtracted. Identification thresholds required ≥2 unique peptide matches per protein with a false discovery rate (FDR) <1%. Nuclear compartment‐enriched proteins were annotated using the DAVID bioinformatics platform v6.7 based on Gene Ontology cellular component terms.^[^ ^56^ ^]^

### Statistical Analysis

All statistical analyses were performed using SPSS software (v20.0). Data were presented as mean ± standard deviation (SD). Fluorescence micrographs and Western blot images were quantified based on integrated density or band intensity, respectively, using ImageJ (NIH), with background subtraction and normalization to loading controls. Comparisons between two groups were conducted using two‐tailed unpaired Student's t‐tests. For multi‐group comparisons, one‐way analysis of variance (ANOVA) followed by Dunnett's post hoc test was applied. All experiments included at least three independent biological replicates, except for RNA‐seq, DIA proteomic sequencing, and IP‐MS analyses, which were performed in duplicate. Statistical significance was defined as *p* < 0.05 (ns: not significant; ^*^ *p* < 0.05, ^**^ *p* < 0.01, ^***^ *p* < 0.001).

## Conflict of Interest

The authors declare no conflict of Interest.

## Author Contributions

S.C.W. and M.H. contributed equally to this work. J.L.H., S.C.W., and M.F.Z. conceived and supervised the project and designed the experiments. S.C.W., M.H. performed most experiments, M.F.Z., W.B.C., and F.D.X. performed the staining experiments. W.P.W., F.D.X., X.L., and C.L.W. were responsible for experimental execution and data collection. W.B.C. and D.H.Y. conducted data organization and analysis. H.J.Z. and H.S.Y. provided critical samples. B.H. and N.L. offered valuable insights. The manuscript was written by S.C.W. and J.L.H. with input and agreement of all authors. All authors read and approved the final version of the manuscript.

## Supporting information



Supporting Information

Supporting Information

## Data Availability

The proteomicsics data that support the findings of this study are openly available in iPROX at https://www.iprox.cn/, reference number IPX0013103001. RNA‐seq data are also openly available in Genome Sequence Archive at https://ngdc.cncb.ac.cn/gsa/, reference number CRA024651.
