# Supplementary material for: BLOC1S1 Attenuates B. Melitensis 16M LPS‐Triggered Autophagy by Spatial Confinement of TDP‐43
Source: Adv Sci (Weinh). 2025 Sep 11;12(45):e05635. doi: 10.1002/advs.202505635 (PMC12677691; doi:10.1002/advs.202505635)
Supplement: Supplementary file 1 — Supporting Information [file ADVS-12-e05635-s002.docx]

**BLOC1S1 Attenuates *Brucella melitensis* LPS-Triggered Autophagy by Spatial Confinement of TDP-43**

Shicheng Wan^1^, Miao Han^1^, Mengfei Zhang, Wenbo Chen, Fangde Xie, Xuan Luo, Wenping Wu, Congliang Wang, Donghui Yang, Bin Han , Haijing Zhu, Haisheng Yu*, Na Li* and Jinlian Hua*

The figures below are uncropped western blotting results.


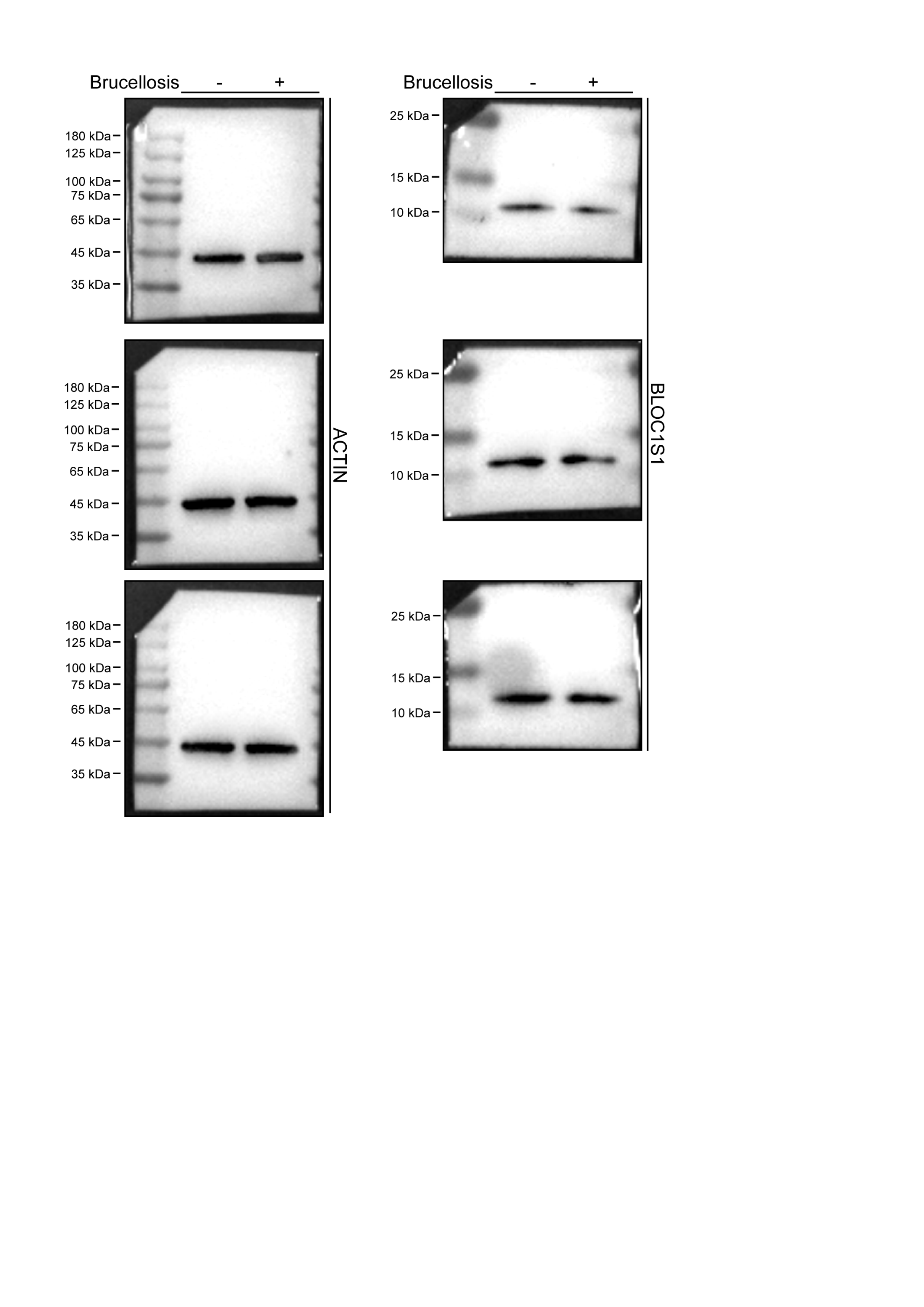


Supplementary Figure 1: Western blotting uncropped blot image in Figure 1I


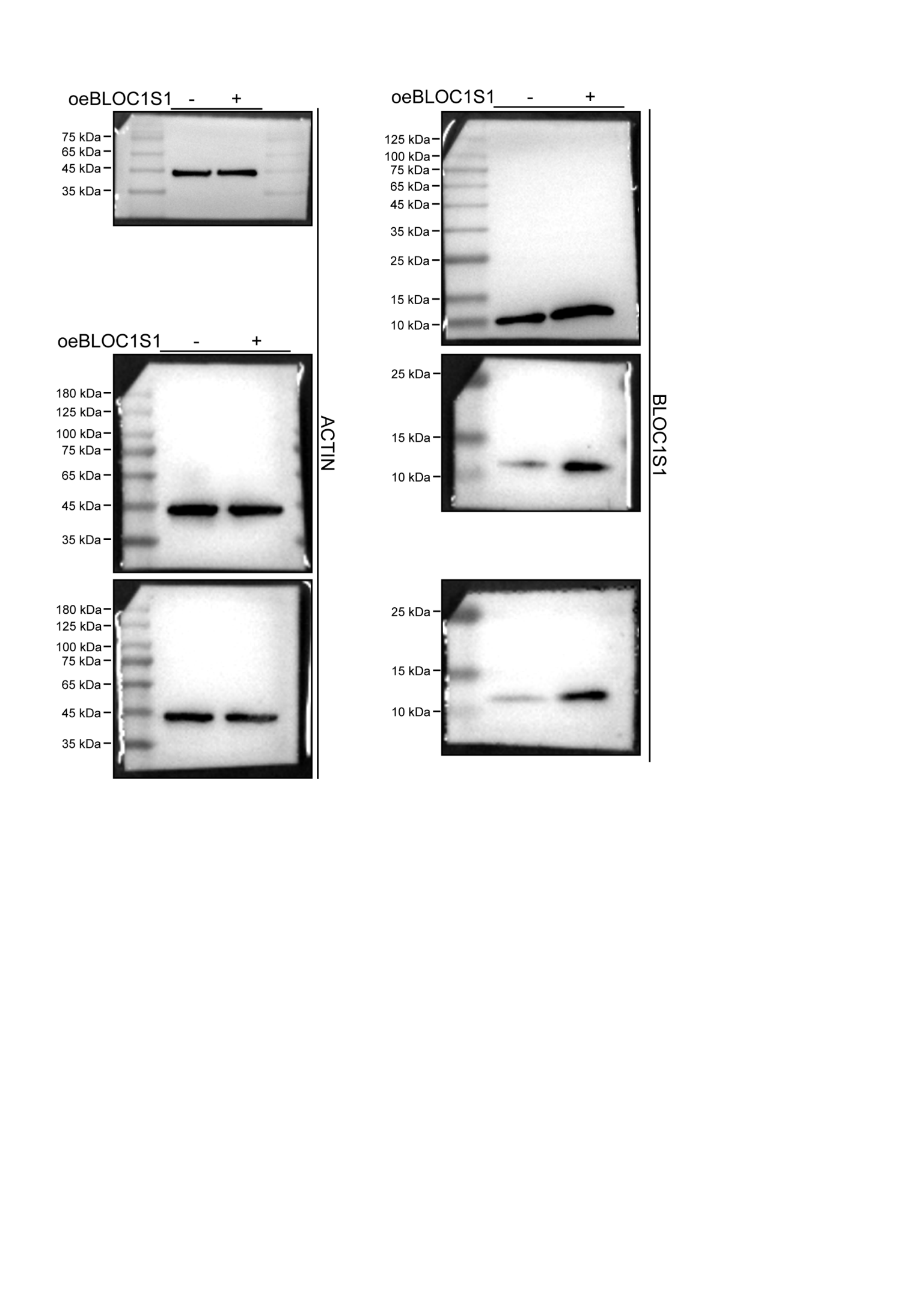


Supplementary Figure 2: Western blotting uncropped blot image in Figure S2D


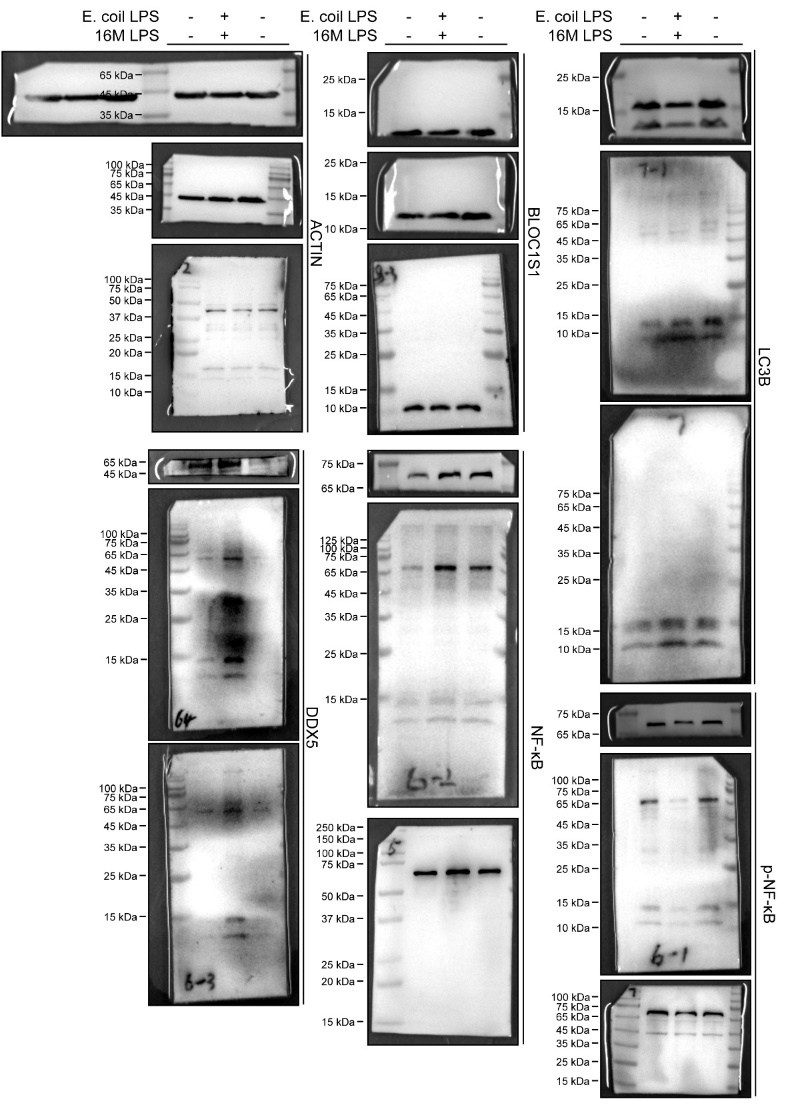

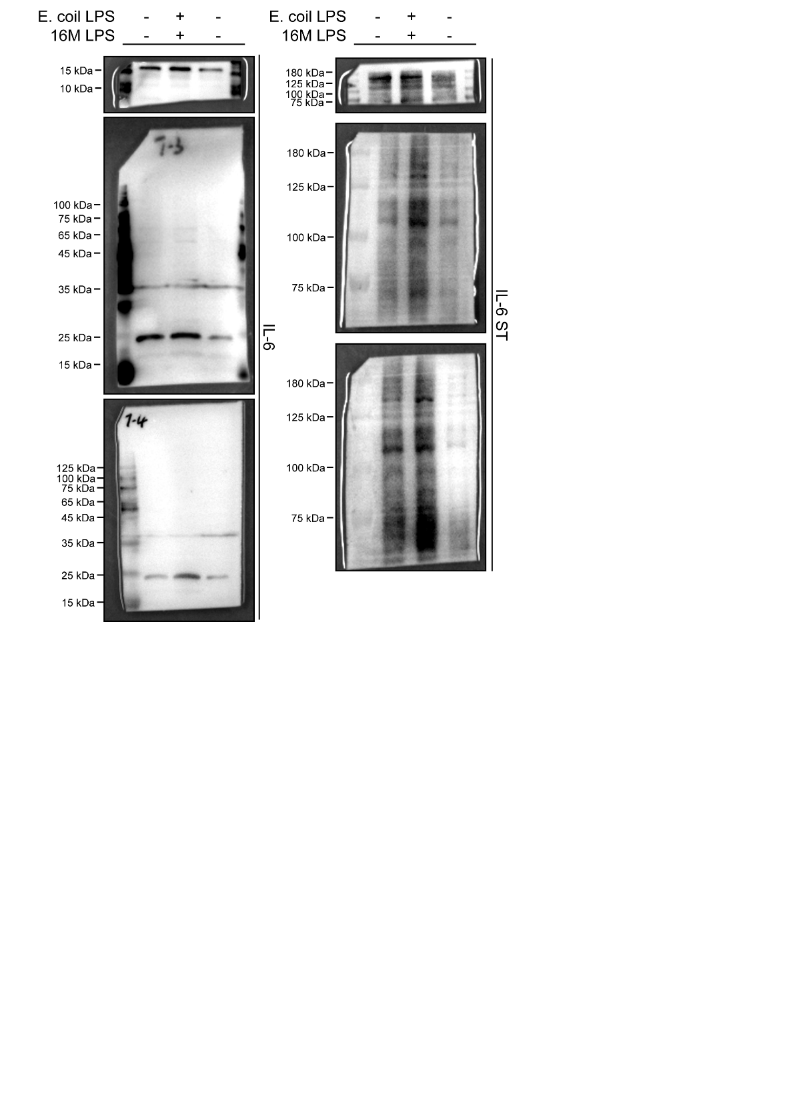
Supplementary Figure 3: Western blotting uncropped blot image in Figure 3F


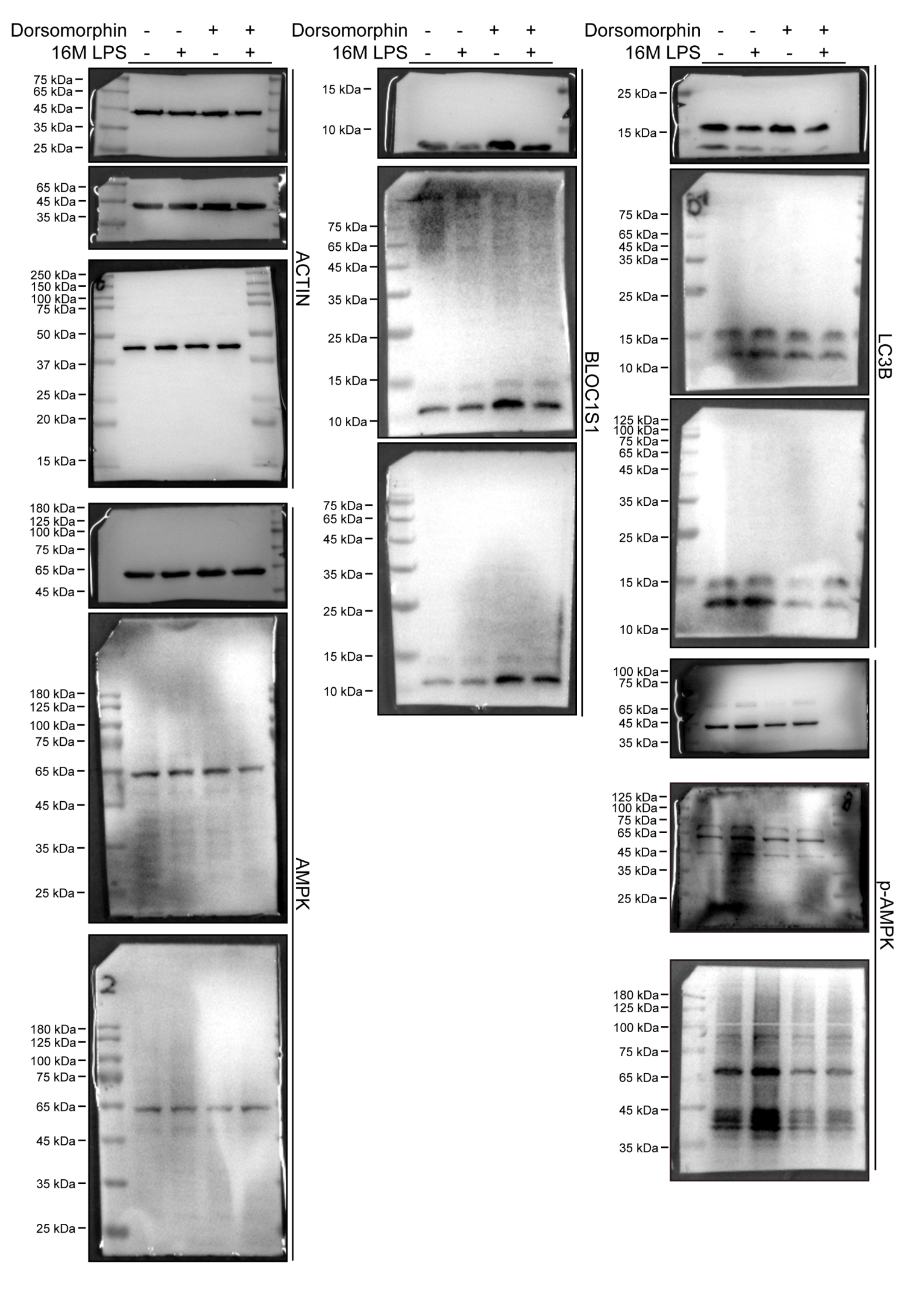


Supplementary Figure 4: Western blotting uncropped blot image in Figure 3H


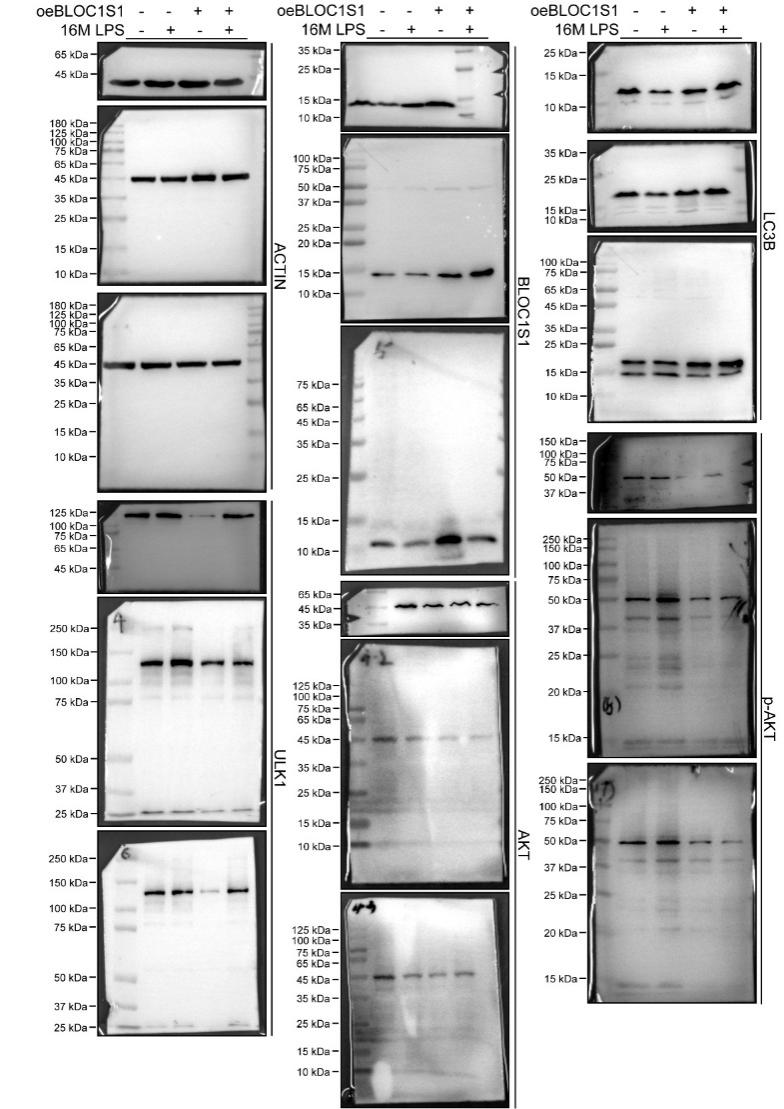

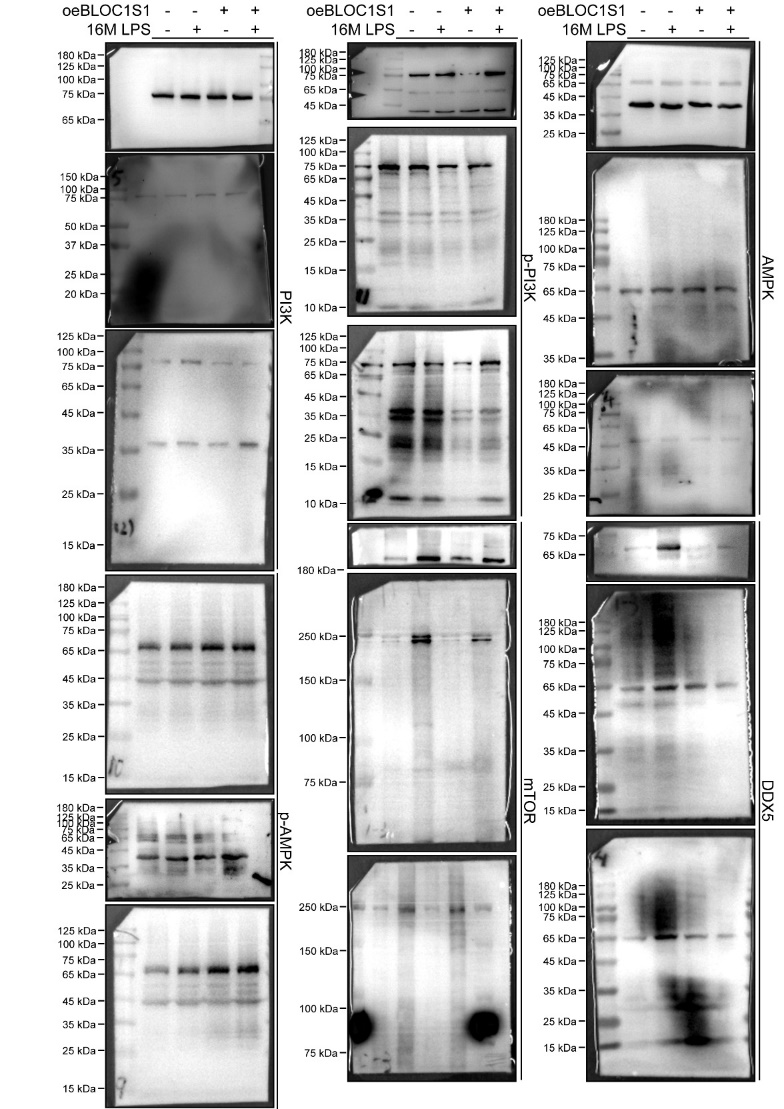

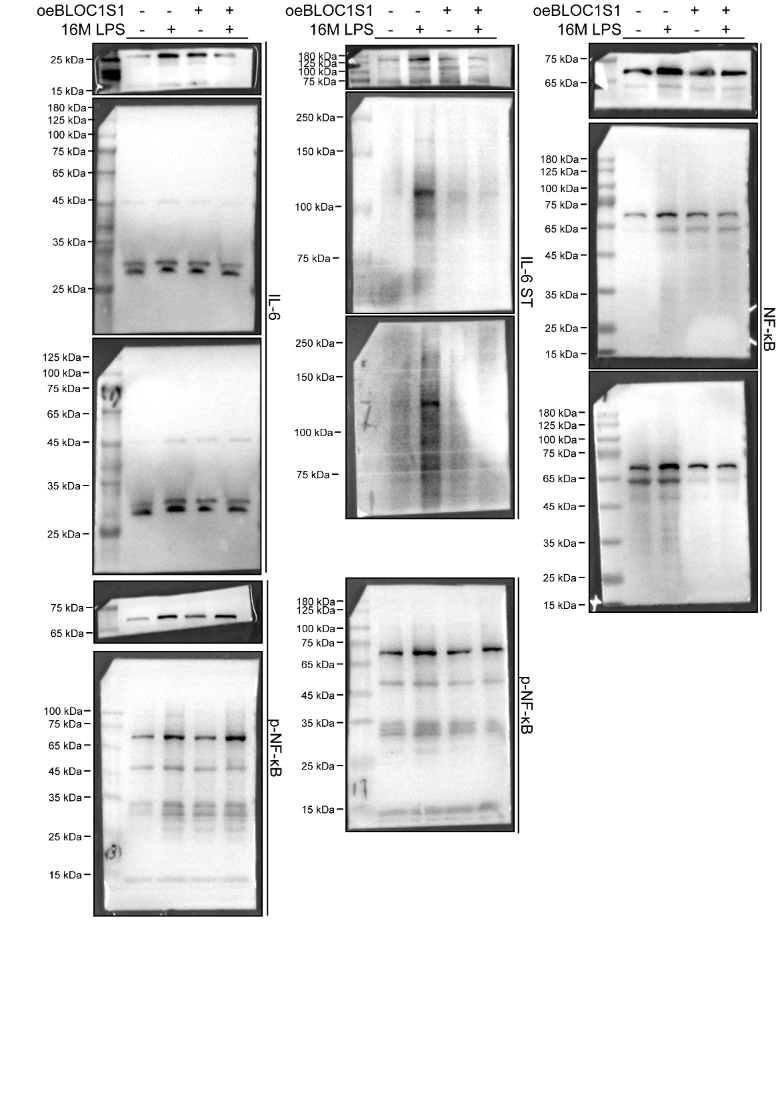


Supplementary Figure 5: Western blotting uncropped blot image in Figure 4D


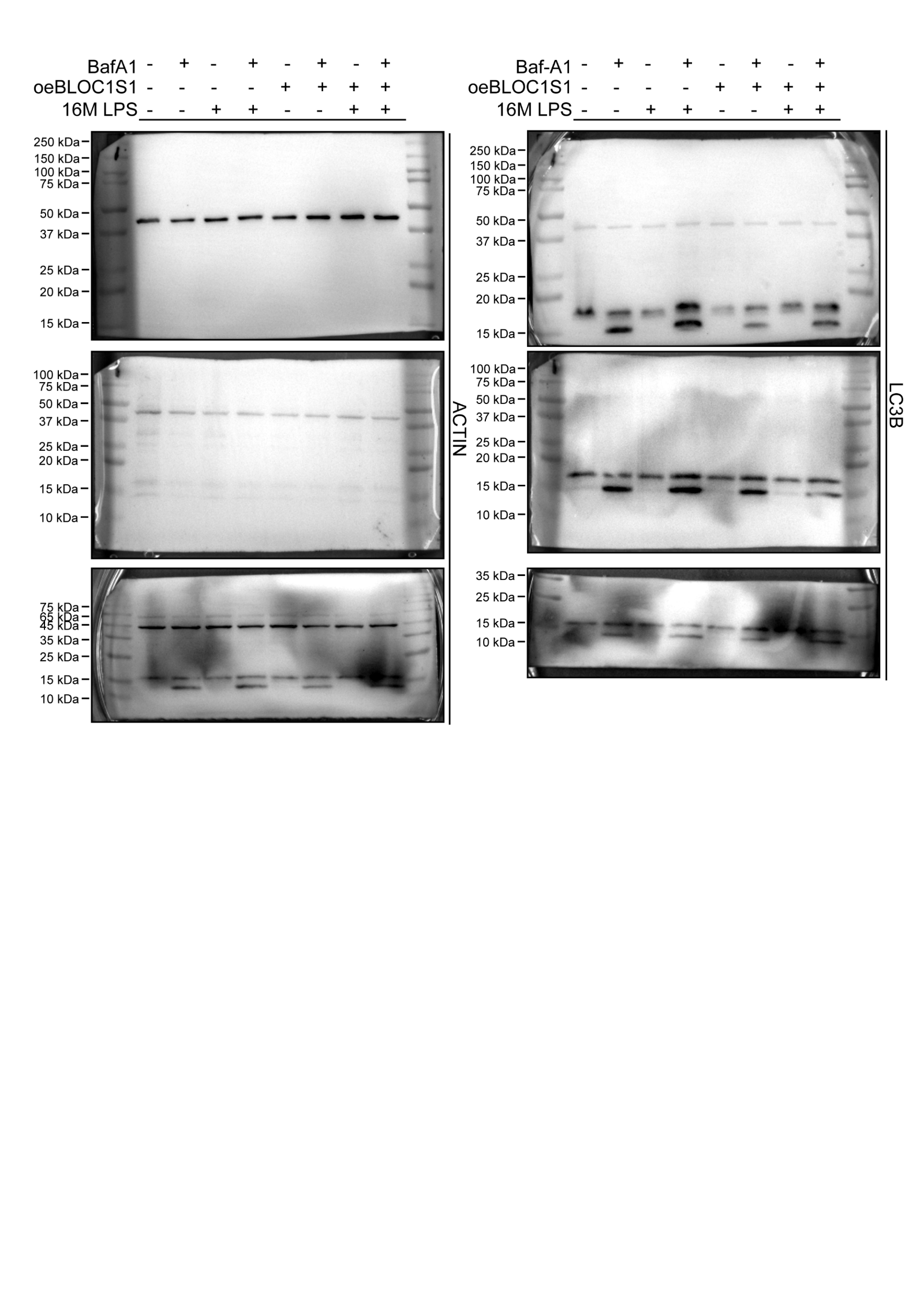


Supplementary Figure 6: Western blotting uncropped blot image in Figure 4E


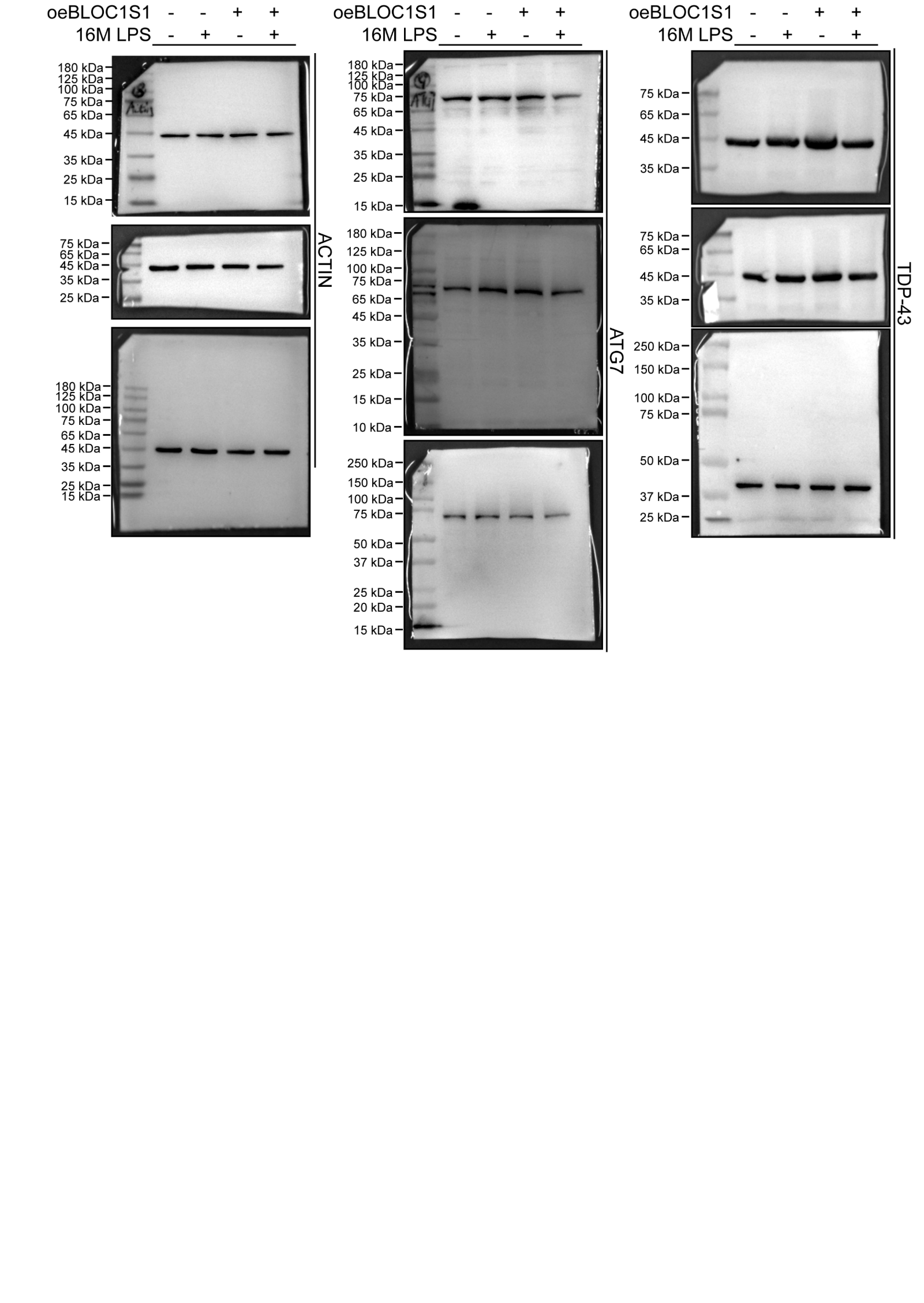


Supplementary Figure 7: Western blotting uncropped blot image in Figure 5G


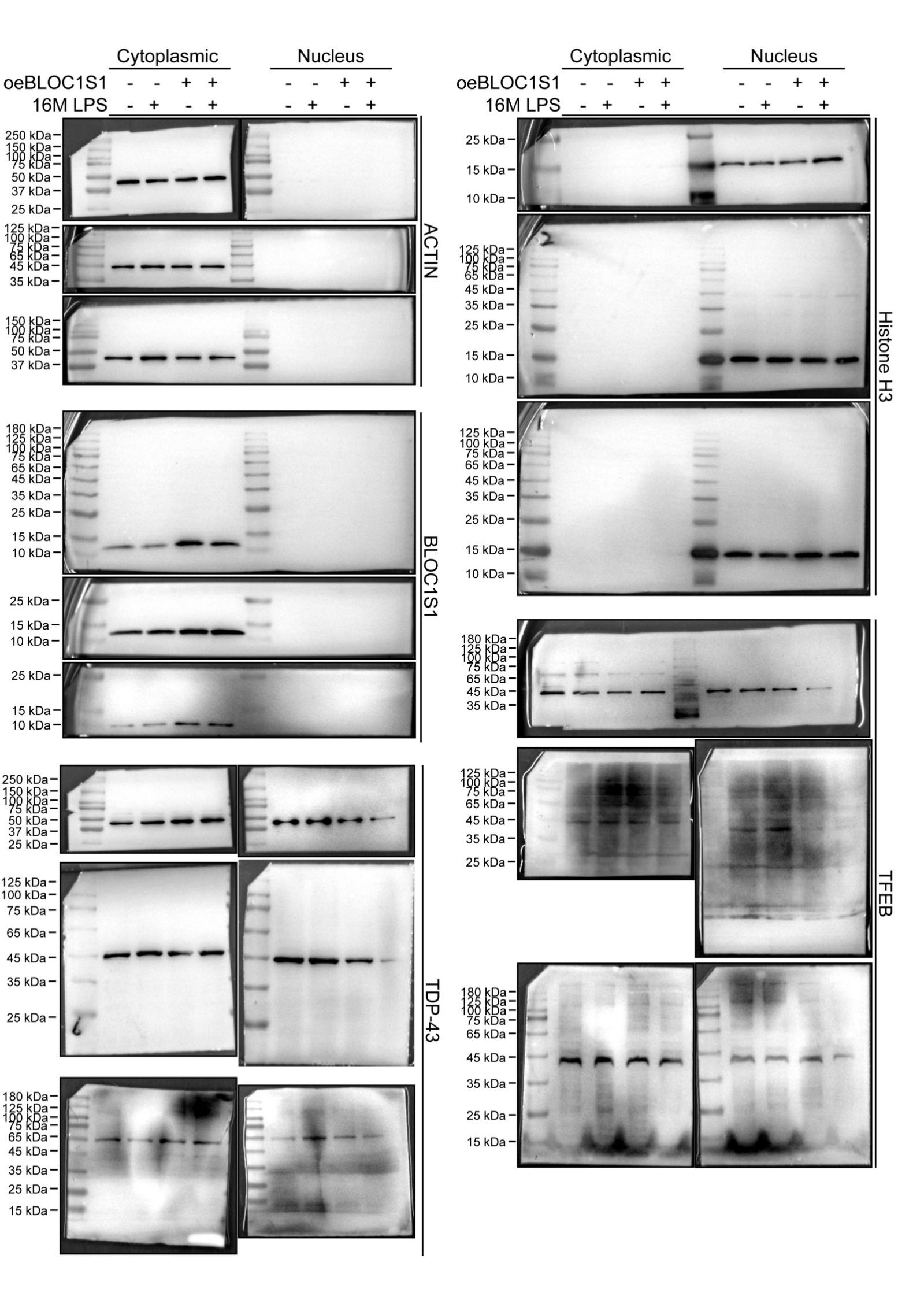


Supplementary Figure 8: Western blotting uncropped blot image in Figure 5H
