# Supplementary material for: BLOC1S1 Attenuates B. Melitensis 16M LPS‐Triggered Autophagy by Spatial Confinement of TDP‐43
Source: Adv Sci (Weinh). 2025 Sep 11;12(45):e05635. doi: 10.1002/advs.202505635 (PMC12677691; doi:10.1002/advs.202505635)
Supplement: Supplementary file 2 — Supporting Information [file ADVS-12-e05635-s001.docx]

**BLOC1S1 Attenuates *B. melitensis* 16M LPS-Triggered Autophagy by Spatial Confinement of TDP-43**

Shicheng Wan^1^, Miao Han^1^, Mengfei Zhang, Wenbo Chen, Fangde Xie, Xuan Luo, Wenping Wu, Congliang Wang, Donghui Yang, Bin Han , Haijing Zhu, Haisheng Yu*, Na Li* and Jinlian Hua*

**This word file includes:**

Figure S1 to Figure S4


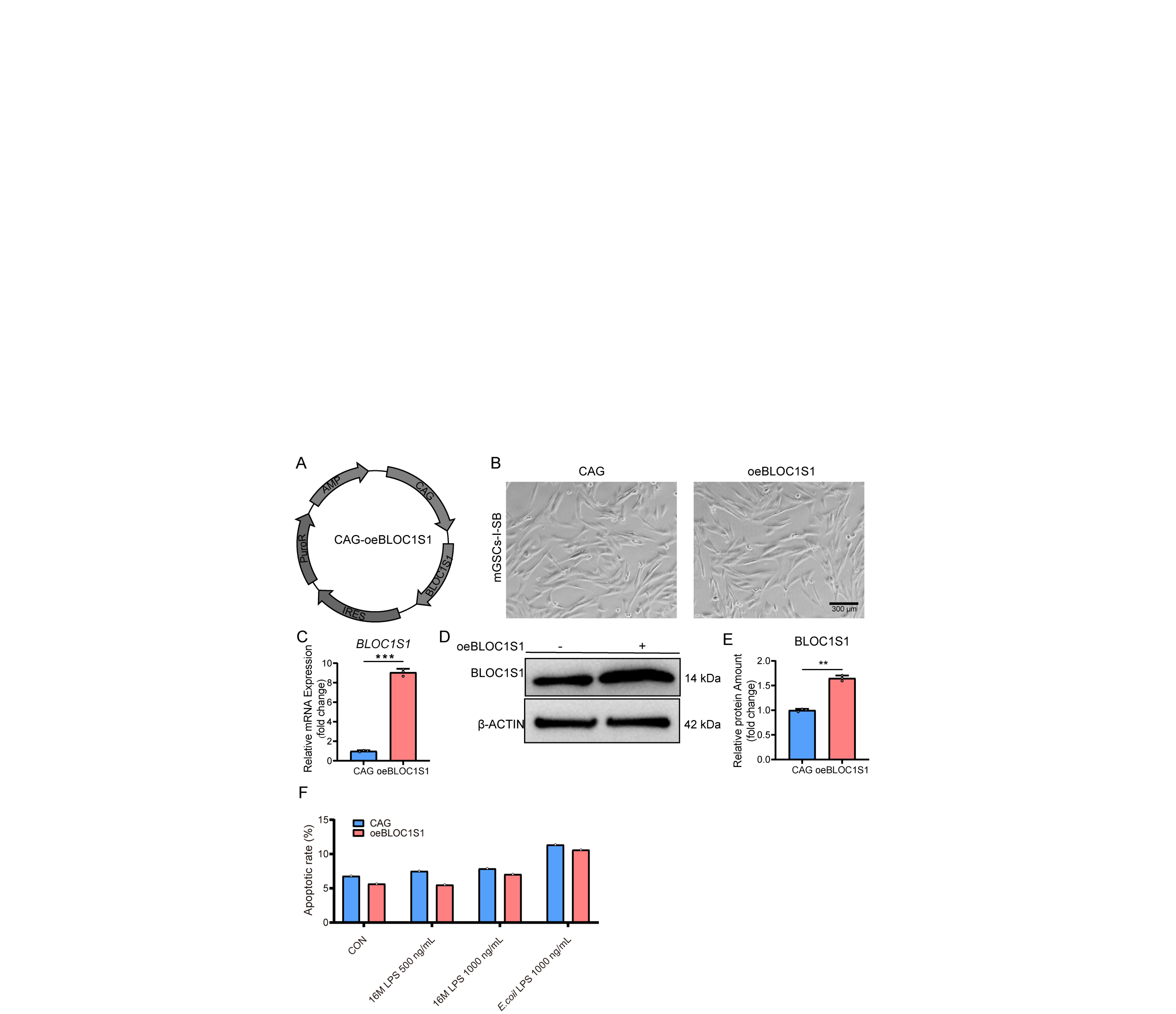


Supplementary Figure S1. Detection of BLOC1S1 overexpression efficiency, related to Figure 2.

A. Schematic representation of the BLOC1S1-overexpressing (oeBLOC1S1) vector.
B. Representative morphology of CAG control, oeBLOC1S1, and mGSCs-I-SB cells. Scale bar: 300 μm.
C. Relative *BLOC1S1* mRNA levels in oeBLOC1S1 versus CAG mGSCs-I-SB cells (*n*=3).
D. BLOC1S1 protein expression in oeBLOC1S1 and CAG mGSCs-I-SB cells.
E. Quantitative analysis of BLOC1S1 protein levels normalized to β-actin (*n*=3).
F. Quantitative analysis of cell apoptosis by flow cytometry as in Figure 3E.

**: *p*<0.01, ***: *p*<0.001.


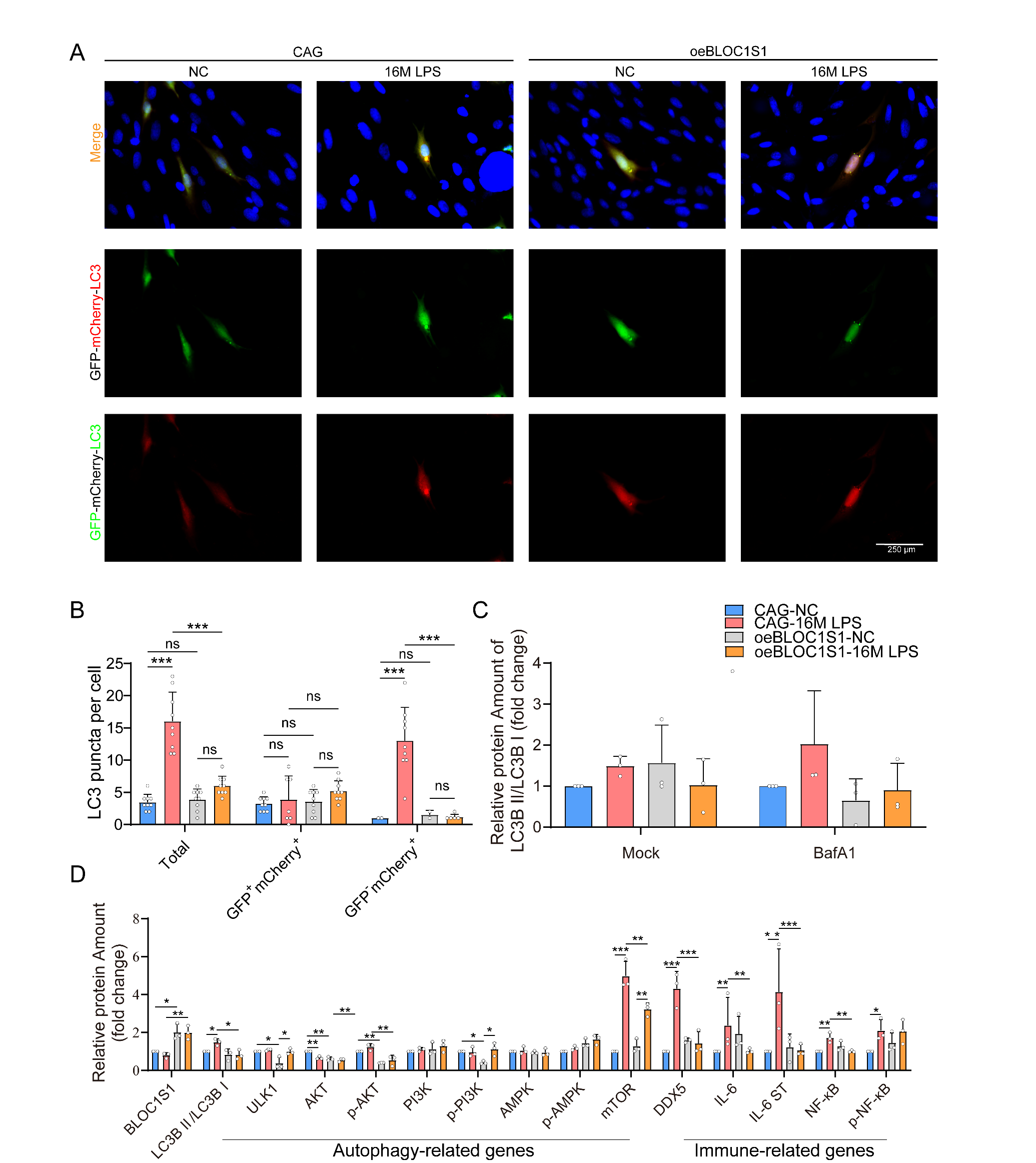


Supplementary Figure S2. Autophagic flux detection and western blotting quantification, related to Figure 4.

A. Representative of GFP-mCherry-LC3 puncta in CAG and oeBLOC1S1 cells. Cells were transiently transfected with plasmid encoding GFP-mCherry-LC3, and then treated with 16M LPS (1 μg/mL, 24 h). The yellow puncta represent autophagosomes (GFP positive and mCherry positive) and red puncta represent autolysosomes (GFP negative and mCherry positive). Scale bar: 200 μm.

B. Quantification of LC3 puncta as in (A).

C: Statistical analysis of the protein amounts of LC3B in oeBLOC1S1 or CAG cells as in Figure 4E. The relative amounts of these proteins were normalized to β-ACTIN.

D. Statistical analysis of the protein amounts of BLOC1S1, LC3B, ULK1, AKT, p-AKT, PI3K, p-PI3K, AMPK. P-AMPK, mTOR, DDX5, NF-κB, phospho-NF-κB, IL-6, IL-6 ST in oeBLOC1S1 or CAG mGSCs-I-SB cells as in Figure 4D, these cells were treated as (Figure 4A). The relative amounts of these proteins were normalized to β-ACTIN.

ns: not significant. *: *p*<0.05, **: *p*<0.01, ***: *p*<0.001.


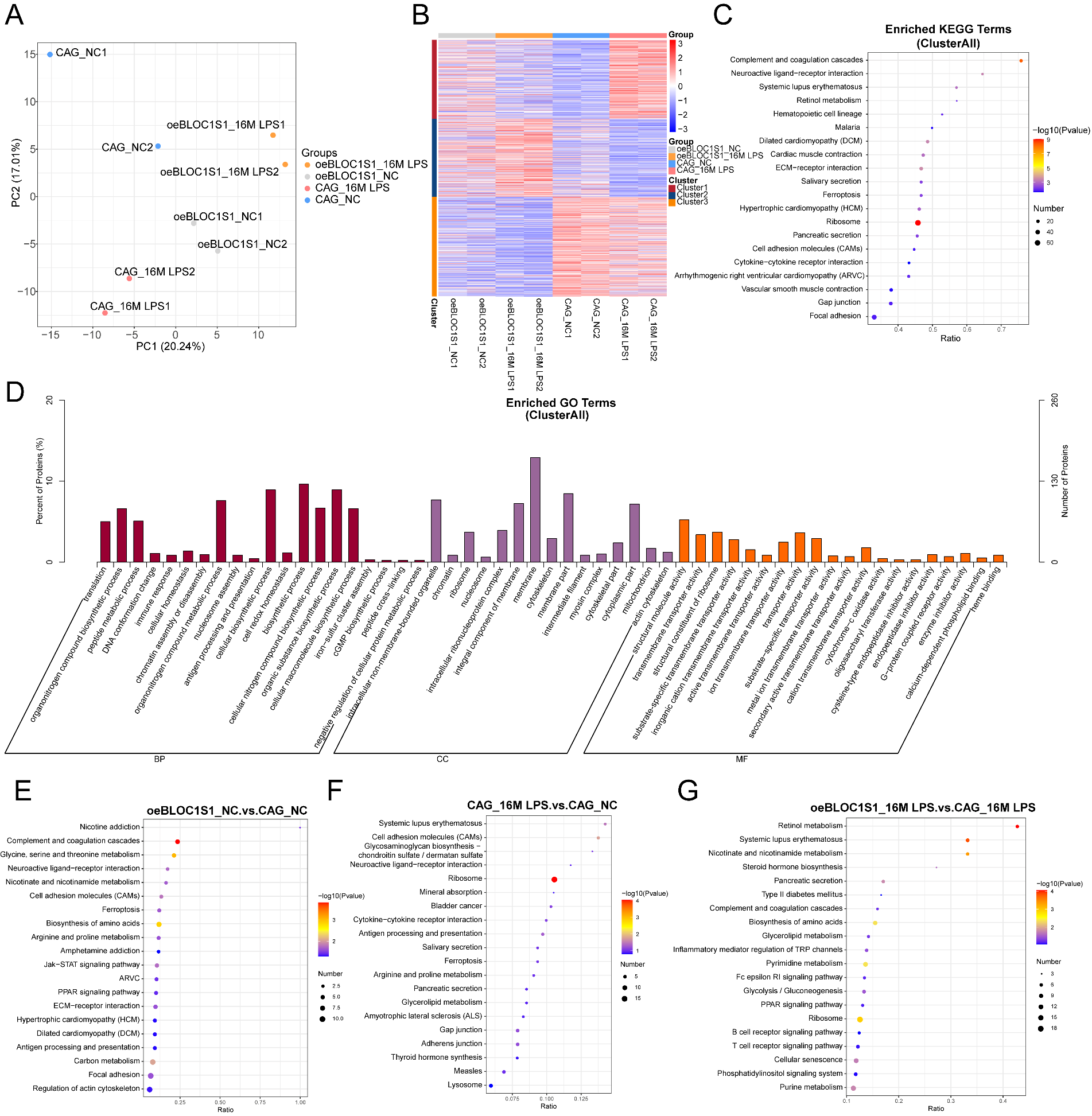


Supplementary Figure S3. DIA proteomic analysis of protein expression alterations induced by 16M LPS treatment, related to Figure 4.

OeBLOC1S1 or CAG mGSCs-I-SB cells were treated with 16M LPS or left untreated for 24 h. Protein samples were collected for sequencing (*n=2*).
A: Principal component analysis (PCA) results;
B: Heatmap showing differentially expressed genes across treatment groups;
C: Top 20 most significantly enriched KEGG pathways (by p-value) for upregulated and downregulated genes in each treatment group;
D: Top 55 most significantly enriched GO biological processes (by *p*-value) for upregulated and downregulated genes across all samples;
E-G: Top 20 most significantly enriched KEGG pathways (by *p*-value) for differentially expressed genes in pairwise comparisons: E) oeBLOC1S1-NC vs CAG-NC; F) CAG-16M LPS vs CAG-NC; G) oeBLOC1S1-16M LPS vs CAG-16M LPS.


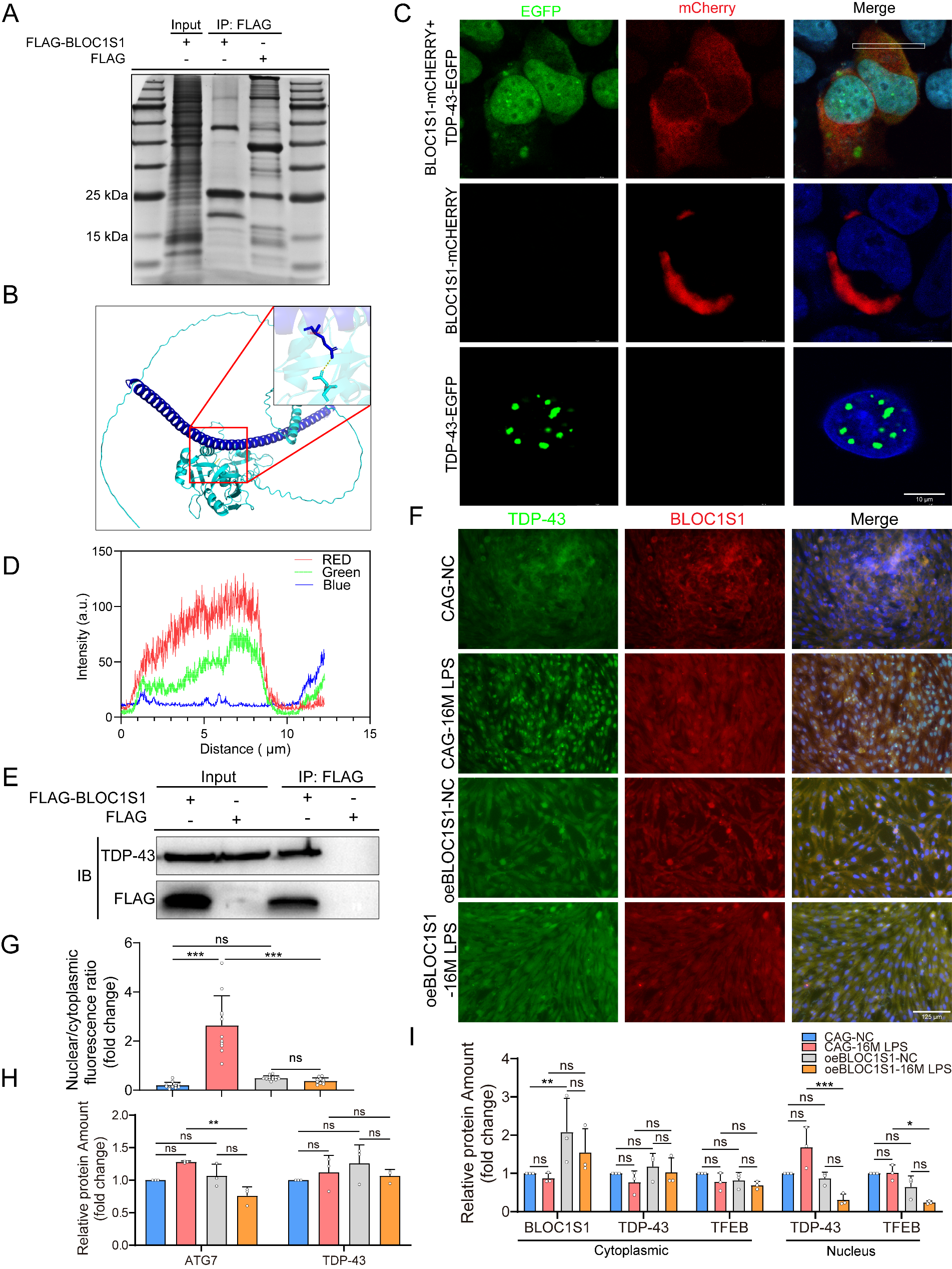


Supplementary Figure S4. BLOC1S1 and TDP-43 are interacting proteins, related to Figure 5.

A: Coomassie brilliant blue staining after electrophoresis of the protein gel precipitated by Co-IP, the mGSCs-I-SB were transfected by FLAG or FLAG-BLOC1S1 fusion protein vectors.

B: Bioinformatics model for predicting interactions between BLOC1S1 and TDP-43 proteins created by Alphafold3, ipTM = 0.15, pTM = 0.23.

C: Intracellular localization of BLOC1S1-mCherry and TDP-43-EGFP in HEK293T. The cells were transfected with two different fusion protein vectors, FALG-BLOC1S1-mCherry and HA-TDP-43-EGFP. Scale bar: 10 μm.

D: Intensity profiles (white square in C) showing signals from all three fluorescent channels.

E: Total lysates were extracted from mGSCs-I-SB transfected by FLAG or FLAG-BLOC1S1 fusion protein vectors for Co-IP experiments. Equal number of proteins was immunoprecipitated separately with Anti-FLAG^®^ M2 Magnetic Beads. Western blots show expression of FLAG and TDP-43 with specific antibodies.

F: IF analysis of CAG, oeBLOC1S1 cells treated with or without 16M LPS for 24 h and stained with antibody against BLOC1S1 (red) and TDP-43 (green). Nucleus were stained with DAPI (blue). Scale bar: 125 μm.

G: Quantification of nuclear-cytoplasmic ratio fluorescence signal of TDP-43 (green) for Figure S4F.

H: Statistical analysis of the protein amounts of TDP-43, ATG7 in oeBLOC1S1 or CAG mGSCs-I-SB cells, these cells were treated as (Figure 5F). The relative amounts of these proteins were normalized to β-ACTIN.

I: Statistical analysis of the protein amounts of BLOC1S1, TDP-43, TFEB in oeBLOC1S1 or CAG mGSCs-I-SB cells, these cells were treated as (F). The relative amounts of these cytoplasmic proteins were normalized to β-ACTIN, and nucleus proteins were normalized to Histone H3.

ns: not significant. *: *p*<0.05, **: *p*<0.01, ***: *p*<0.001.
